# Lateral distribution of endometriotic lesions: the anatomical recesses hypothesis. A systematic review and meta-analysis

**DOI:** 10.1093/hropen/hoaf064

**Published:** 2025-10-24

**Authors:** Veronica Bandini, Sonia Cipriani, Chiara Pillinini, Stefano Angioni, Ludovica Bartiromo, Anna Biasioli, Massimo Candiani, Valerio Carletti, Maurizio Nicola D’Alterio, Domenico Incandela, Lucia Lazzeri, Antonio Maiorana, Ludovico Muzii, Alessio Perandini, Federica Perelli, Maria Grazia Porpora, Diego Raimondo, Valentino Remorgida, Giulia Rovero, Federica Savasta, Stefano Scarperi, Matteo Schimberni, Renato Seracchioli, Eugenio Solima, Giuseppe Sorrenti, Roberta Venturella, Michele Vignali, Giuseppe Vizzielli, Veronica Yacoub, Fulvio Zullo, Errico Zupi, Paolo Vercellini, Endometriosis Treatment Italian Club

**Affiliations:** Academic Centre for Research on Adenomyosis and Endometriosis, Department of Clinical Sciences and Community Health, Università degli Studi di Milano, Milan, Italy; Gynecology Unit, Fondazione IRCCS Ca’ Granda Ospedale Maggiore Policlinico, Milan, Italy; Academic Centre for Research on Adenomyosis and Endometriosis, Department of Clinical Sciences and Community Health, Università degli Studi di Milano, Milan, Italy; Division of Gynecology and Obstetrics, Department of Surgical Sciences, University of Cagliari, Cagliari, Italy; Gynecology/Obstetrics Unit, IRCCS San Raffaele Scientific Institute, Milan, Italy; Clinic of Obstetrics and Gynecology, Santa Maria della Misericordia University Hospital, Udine, Italy; Gynecology/Obstetrics Unit, IRCCS San Raffaele Scientific Institute, Milan, Italy; Department of Obstetrics and Gynecology, University of Rome Tor Vergata, Rome, Italy; Division of Gynecology and Obstetrics, Department of Surgical Sciences, University of Cagliari, Cagliari, Italy; Obstetrics and Gynecology Unit, ARNAS Civico Di Cristina Benfratelli, Palermo, Italy; Department of Molecular and Developmental Medicine, Obstetrics, Gynecological Clinic, University of Siena, Siena, Italy; Obstetrics and Gynecology Unit, ARNAS Civico Di Cristina Benfratelli, Palermo, Italy; Department of Maternal and Child Health and Urological Sciences, University of Rome La Sapienza, Rome, Italy; Obstetrics and Gynecology Division B, University of Verona, Verona, Italy; Department of Women and Child Health, Azienda USL Toscana Centro, Florence, Italy; Pediatric Gynecology Unit, Meyer Children’s Hospital, Florence, Italy; Department of Maternal and Child Health and Urological Sciences, University of Rome La Sapienza, Rome, Italy; Division of Gynecology and Human Reproduction Physiopathology, IRCCS Azienda Ospedaliero-Universitaria di Bologna Policlinico di Sant’Orsola, Bologna, Italy; Department of Gynecology and Obstetrics, University Hospital Maggiore della Carità, Novara, Italy; Department of Women and Child Health, Azienda USL Toscana Centro, Florence, Italy; Department of Gynecology and Obstetrics, University Hospital Maggiore della Carità, Novara, Italy; Obstetrics and Gynecology Division B, University of Verona, Verona, Italy; Gynecology/Obstetrics Unit, IRCCS San Raffaele Scientific Institute, Milan, Italy; Division of Gynecology and Human Reproduction Physiopathology, IRCCS Azienda Ospedaliero-Universitaria di Bologna Policlinico di Sant’Orsola, Bologna, Italy; Department of Biomedical Science for Health, Università degli Studi di Milano, Milan, Italy; Department of Gynecology, San Carlo Nancy Hospital, Rome, Italy; Department of Experimental and Clinical Medicine, Magna Graecia University of Catanzaro, Catanzaro, Italy; Department of Biomedical Science for Health, Università degli Studi di Milano, Milan, Italy; Clinic of Obstetrics and Gynecology, Santa Maria della Misericordia University Hospital, Udine, Italy; Department of Obstetrics and Gynecology, University of Rome Tor Vergata, Rome, Italy; Department of Experimental and Clinical Medicine, Magna Graecia University of Catanzaro, Catanzaro, Italy; Department of Molecular and Developmental Medicine, Obstetrics, Gynecological Clinic, University of Siena, Siena, Italy; Academic Centre for Research on Adenomyosis and Endometriosis, Department of Clinical Sciences and Community Health, Università degli Studi di Milano, Milan, Italy; Gynecology Unit, Fondazione IRCCS Ca’ Granda Ospedale Maggiore Policlinico, Milan, Italy

**Keywords:** endometriosis, ovary, uterosacral ligament, colon, ureter, inguinal region, diaphragm, pleura, lung

## Abstract

**STUDY QUESTION:**

Are endometriotic lesions affecting bilateral organs or anatomical structures distributed symmetrically on both sides of the body?

**SUMMARY ANSWER:**

The left-sided preponderance of pelvic endometriotic lesions, and the right-sided dominance of thoracic and inguinal lesions, suggest that endometriotic lesions exhibit a non-random, asymmetric lateral distribution.

**WHAT IS KNOWN ALREADY:**

Evaluating the anatomical distribution of endometriotic lesions may elucidate the underlying pathogenic mechanism(s) of the disease. If the coelomic metaplasia or embryonic cell remnant theory is correct, a symmetrical right-left pattern would be expected. Conversely, retrograde menstruation would likely result in asymmetrical distribution, influenced by gravity, peritoneal fluid circulation, and anatomical niches.

**STUDY DESIGN, SIZE, DURATION:**

This systematic review with meta-analysis included full-length, English-language articles published up to 10 June 2024. Literature searches were performed in PubMed/Medline and Embase databases with the keyword ‘endometriosis’, ‘lateral’, ‘distribution’, ‘right’, ‘left’, and ‘asymmetry’.

**PARTICIPANTS/MATERIALS, SETTING, METHODS:**

The review focused on anatomical structures commonly affected by endometriosis with surgically defined right or left laterality: ovaries, uterosacral ligaments, colon, ureters, inguinal regions, and hemithorax (diaphragm, pleura, lungs). Case reports were excluded. Risk of bias was assessed using ROBINS-I for non-randomized studies and a dedicated tool for case series. Meta-analyses of proportions were conducted in R. Heterogeneity was quantified using the *I*^2^ statistic. Funnel plots for publication bias and Egger tests were performed using Stata.

**MAIN RESULTS AND THE ROLE OF CHANCE:**

Of 6356 articles screened, 154 met the inclusion criteria. A statistically significant left-sided preponderance was observed for ovarian (58%; 95% CI: 57–60%; *P < *0.001), uterosacral ligament (56%; 95% CI: 54–59%; *P < *0.001), ureteral (71%; 95% CI: 67–76%; *P < *0.001), and bowel (72%; 95% CI: 64–79%; *P < *0.001) lesions, whereas thoracic (98%; 95% CI: 96–100%; *P < *0.001) and inguinal (92%; 95% CI: 83–98%; *P < *0.001) lesions were predominantly right-sided. These findings were confirmed in the sensitivity analyses. Egger’s test indicated a possible small study effect only for ovarian lesions (*P *= 0.012).

**LIMITATIONS, REASONS FOR CAUTION:**

The preponderance of retrospective studies, the variability in surgical procedures, and the potential difficulties in accurately distinguishing unilateral from bilateral lesions may have influenced the magnitude of the estimated difference. However, the large patient cohorts, geographical diversity, and consistent asymmetry across lesion types strengthen the results’ validity and generalizability.

**WIDER IMPLICATIONS OF THE FINDINGS:**

The pattern of endometriotic lesion distribution, including the opposite asymmetry observed in the pelvis and upper abdomen/thorax, can be explained by factors influencing dissemination and implantation of refluxed endometrial cells. However, it cannot be explained as well by the coelomic metaplasia or embryonic cell remnant theories. This may have important clinical implications, providing a pathogenic basis for secondary prevention strategies.

**STUDY FUNDING/COMPETING INTEREST(S):**

The open access facility of this paper was funded by the Italian Ministry of Health, Current research IRCCS Ca’ Granda Ospedale Maggiore Policlinico. P.V. is a member of the Editorial Board of *Human Reproduction Open*, *Journal of Obstetrics and Gynaecology Canada*, and International Editorial Board of *Acta Obstetricia et Gynecologica Scandinavica*; has received royalties from Wolters Kluwer for chapters in UpToDate. All other authors declare no conflicts of interest.

**REGISTRATION NUMBER:**

CRD42024511356 (PROSPERO).

WHAT DOES THIS MEAN FOR PATIENTS? We reviewed published studies to verify whether endometriosis develops equally on both sides of the body or if one side is more frequently affected. We found that endometriosis is more common in the left side of the pelvis, but it is more often found on the right side of the chest and inguinal region. Understanding these patterns may help doctors learn more about how endometriosis develops. This knowledge can support earlier diagnosis and improve strategies for preventing disease progression.

## Introduction

Despite the high prevalence, the associated individual and societal burden, and the intense research efforts over the past decades, the exact pathogenesis of endometriosis remains elusive (Giudice and Kao, 2004; Bulun, 2009; Vercellini *et al.*, 2014; Bulun *et al.*, 2019; Zondervan *et al.*, 2020; Taylor *et al.*, 2021; Horne and Missmer, 2022; Endometriosis Initiative Group, 2024; As-Sanie *et al.*, 2025; Garcia-Velasco *et al.*, 2025). Although disease promotion and progression are likely to be multifactorial, involving, among other factors, genetic, epigenetic, hormonal, microbiological, and environmental influences (Koninckx *et al.*, 2019; Laganà *et al.*, 2019), two general pathogenic alternatives, i.e. *in situ* ectopic development of endometrial epithelial and stromal cells (Lauchlan, 1972; Redwine, 1987, 1988; Fujii, 1991; Donnez *et al.*, 1997; Taylor and Williams, 2010) versus migration from the uterine cavity and secondary abdominal implantation, are recognized for disease initiation. The former scenario includes the coelomic metaplasia and the embryonic cell rest theories, whereas the latter is based on the retrograde menstruation theory (Nisolle and Donnez, 1997; Irving and Clement, 2019).

An indirect way of trying to unravel which of the above theories is true is to assess the anatomical distribution of endometriotic lesions. In their seminal article published 40 years ago, Jenkins *et al.* (1986) adopted a kind of ‘sagittal view’ of the pelvis, focusing on the unequal frequency of endometriotic lesions in the antero- and postero-uterine compartments, possibly determined by uterine flexion, the creation of dependent peritoneal pockets facilitating the collection of refluxed endometrium, and the location of the tubal fimbriae.

Studying the lateral distribution of endometriotic lesions on bilateral anatomical structures would test the different hypotheses even more reliably, since a considerable asymmetry would appear biologically incompatible with both the coelomic metaplasia and the embryonic cell rest theories. Contrariwise, the retrograde menstruation theory would be compatible with the metastasis of refluxed endometrial fragments according to gravity, physiological determinants of abdominal fluid flow, and specific anatomical features of the receptor sites that facilitate implantation differently on either side (Jenkins *et al.*, 1986; Chapron *et al.*, 2006; Vercellini *et al.*, 2007; Bricou *et al.*, 2008).

Such lateral asymmetry was observed by several independent research groups in the late 1990s and early 2000s (Vercellini *et al.*, 1998,^ 2002^; Chapron *et al.*, 2001; Ghezzi *et al.*, 2001; Al-Fozan and Tulandi, 2003; Parazzini, 2003; Sznurkowski and Emerich, 2008; Bazi *et al.*, 2007), but has never been mentioned in the reviews published during the past two decades in the four highest-ranking general medicine journals (Giudice and Kao, 2004; Bulun, 2009; Zondervan *et al.*, 2020; Taylor *et al.*, 2021; Horne and Missmer, 2022; As-Sanie *et al.*, 2025).

Two literature reviews on the anatomical distribution of endometriotic lesions were published several years ago, but the conclusions were partly inconsistent (Bricou *et al.*, 2008; Guo *et al.*, 2008). Moreover, a high between-study heterogeneity of proportions was observed, which was strongly influenced by the sample size (Guo *et al.*, 2008). Therefore, the members of the Endometriosis Treatment Italian Club (ETIC), comprising investigators from 15 university departments and teaching hospitals in Italy, deemed it relevant to try to definitively clarify whether unilateral endometriotic lesions detected at surgery on bilateral anatomical structures/organs are distributed asymmetrically.

## Methods

The study protocol was registered with PROSPERO (registration ID number CRD42024511356). The systematic review with meta-analysis was conducted according to the Preferred Reporting Items for Systematic Reviews and Meta-Analyses (PRISMA) guideline (Page *et al.*, 2021) and was exempt from ethical approval because only published and de-identified data were used. The review focused on those anatomical structures/organs most commonly affected by endometriosis and for which a right and left side can be unambiguously determined, namely the ovaries, uterosacral ligaments (USLs), colon, ureters, inguinal regions, and hemithorax (including the diaphragm, pleura, and lung). Inclusion of superficial peritoneal endometriotic lesions in the pelvis was not deemed feasible, as laterality is generally not clearly defined. Furthermore, even when anatomically described, peritoneal lesions are frequently not histologically confirmed (Moen and Halvorsen, 1992; Walter *et al.*, 2001; Marchino *et al.*, 2005; Kazanegra *et al.*, 2008; Fernando *et al.*, 2013).

### Search strategy and inclusion criteria

For each participating centre, we identified two reviewers who independently conducted the literature screening following the same strategy but targeting different lesion sites. To identify eligible studies, the PubMed/Medline and Embase electronic databases were searched from inception to 10 June 2024 using the term ‘endometriosis’ in combination with ‘lateral’, ‘distribution’, ‘right’, ‘left’, ‘asymmetry’, and additional keywords to widen the search for potentially relevant studies. The search terms and the algorithm used are described in detail in Supplementary File S1. In addition, the reference lists of the retrieved articles were systematically examined manually, and the ‘similar articles’ and ‘cited by’ functions of PubMed were used to identify further relevant publications.

This review was restricted to full-length, English-language articles published in peer-reviewed journals. All reports describing the lateral distribution of endometriotic lesions affecting the above structures were considered, regardless of the study design, except for case reports, which were excluded from the meta-analyses.

Articles were included if endometriotic lesions had been biopsied or surgically removed. Some studies without a histological diagnosis, mainly on diaphragmatic/thoracic endometriosis, were selected only if typical symptoms were present and characteristic lesions were visualized intraoperatively. Studies in which the laterality of the endometriotic lesions was assessed only by ultrasound were excluded, as were studies in which the lesions were described as unilateral or bilateral but without specifying how many lesions were located on the right versus the left side. Studies involving pregnant patients, those with only histologically atypical endometriosis, or those with concomitant pelvic inflammatory disease were also excluded.

Regarding the colon, left laterality was determined as lesions in the sigmoid and descending colon, whereas right laterality was attributed to lesions in the ascending colon, cecum, ileocecal junction, terminal ileum, and appendix. Endometriosis of the rectum, recto-sigmoid junction, transverse colon, and the remaining parts of the small bowel were excluded from the analysis because these locations cannot be clearly and systematically attributable to either side. For example, recto-sigmoidal involvement could be classified as left-sided, but this can be misleading, as lesions of this tract usually arise from implants in the pouch of Douglas, therefore representing a primarily midline localization (Vercellini *et al.*, 2000a, 2004). Studies on colon endometriosis were included if it was stated that the entire large bowel was assessed, allowing the identification of left, right, or multiple lesions. A comparison conducted in a population in which both sides have been adequately assessed would be less susceptible to a potential detection bias introduced by inconsistent evaluation of the ascending colon and a selection bias, because studies focusing exclusively on the sigmoid colon might overestimate the prevalence of left-sided lesions. Therefore, articles that exclusively considered a single bowel segment (e.g. sigmoid, cecum, or appendix) were excluded.

Concerning thoracic endometriosis, all studies that assessed the laterality of diaphragmatic, pleural, and pulmonary lesions, alone or in combination, were included in the meta-analysis. Rather than analysing each site separately, all thoracic sites were combined because diaphragmatic lesions often coexist with pleural and/or pulmonary involvement and vice versa. Moreover, the lateral distribution of these lesions is not always reported separately. Except for two studies published in 1998 in which histological confirmation of the lesions was provided (Flieder *et al.*, 1998 and Nezhat *et al.*, 1998), we selected studies published from 2000 onward for this anatomic compartment to ensure greater consistency and reliability, as thoracoscopic techniques were not systematically implemented in the last century (Channabasavaiah and Joseph, 2010). Consequently, cases with histologically confirmed endometriosis were included, as well as cases of thoracic endometriosis diagnosed based on typical symptoms (e.g. catamenial pneumothorax) together with intraoperative visualization of characteristic lesions by video-assisted thoracic surgery in patients with concomitant or previously documented pelvic endometriosis.

### Study selection and data extraction

Two reviewers from each participating centre independently screened publications by examining the titles and abstracts related to the right or left distribution of specific endometriotic lesions in patients undergoing surgery. Eligibility was then assessed by retrieving the selected relevant full-text articles. Disagreements were resolved by discussion with V.B. and C.P. from the coordinating centre.

The following data were extracted from each report: first author’s surname, year of publication, country in which the research was conducted, study design, mean age of participants, number of patients with right lesions, number of patients with left lesions, and number of patients with bilateral lesions. In addition, the type of surgery performed was reported for the USL, ureter, bowel, and inguinal region. For thoracic endometriosis, the exact location within the chest cavity (pleura, lung, diaphragm) was described. Data were collected for all the selected studies, including those in which the location of the lesion was not explicitly specified as being unilateral or bilateral. In fact, in some of the articles, it was not clearly stated whether the lesions in the target population were only unilateral or whether some patients could have bilateral lesions. As the aim of our study was to assess whether the laterality of lesions was equally distributed between the right and left sides, we first calculated the proportion of right (or left) lesions in the total population, including patients in whom the unilateral versus bilateral status was unknown, and then performed the same analysis considering only articles in which unilateral lesions were explicitly reported. However, when it was clear how many patients had unilateral lesions and how many had bilateral lesions, we did not consider the latter subgroup because here the proportion of laterality is necessarily equal to 1 (100%) for both the right and the left side. This adds no information with respect to the purpose of the study. Estimating laterality, including bilateral lesions, would mean adding right (or left) lesions to both the numerator and the denominator, which is mathematically inappropriate and redundant for the above considerations.

All the individual reports from the participating centres were reviewed by V.B. and C.P., who supervised the entire search strategy, performed an independent overall literature search, and checked the completeness of the article retrieval and the correctness of the data extraction. The global accuracy of the process was checked by S.C., a senior methodologist, through periodic sampling.

### Quality assessment and risk of bias

The quality of the included studies and the potential risk of bias were evaluated using the Risk Of Bias In Non-randomized Studies of Interventions (ROBINS-I) tool (Sterne *et al.*, 2016) for cohort, case-control and cross-sectional studies and the synthesis tool for evidence derived from case reports and case series, developed by Murad *et al.* (2018) for case series included in the meta-analysis. This tool assesses four areas of potential bias: selection, ascertainment, causation, and reporting. It consists of eight core explanatory questions, each of which requires a binary response, resulting in a potential total score of 0–8. However, questions 5 and 6 have not been considered here, as the ‘challenge/rechallenge phenomenon’ and the ‘dose-response effect’ were deemed irrelevant to the present study. Consequently, the overall rating ranges from 0 to 6.

Assessment and rating were performed independently by two reviewers (V.B. and C.P.). Any disagreement in the appraisal of the certainty of the evidence was resolved by consultation with a third reviewer (S.C.) until a unanimous decision was reached.

### Data synthesis and meta-analysis

All analyses were performed using Metaprop, a command implemented in R, for calculating meta-analyses of proportions. Estimates of proportions (percentages) and 95% CIs were calculated using a random effects model. The Freeman–Tukey double arcsine square root transformation was applied to the data before pooling for meta-analysis. This transformation stabilizes the variance and allows proportions equal to zero or one to be included in the calculation. The hypothesis to be tested was an equal distribution between the left and the right side. Therefore, the null hypothesis was a proportion of laterality equal to 0.5. With this assumption, a probability value <0.05 means that the laterality estimated by the meta-analysis is significantly different from 0.5 (50%).

Furthermore, the *I*^2^ value was calculated to assess the quantitative heterogeneity between studies. In cases where *I^2^* was significant, heterogeneity was investigated by performing subgroup analyses (retrospective studies vs prospective studies) or with sensitivity analyses (e.g. considering only studies that explicitly documented unilateral lesions or the specific surgical procedures accomplished). All these analyses were performed using R software, version 4.4.2 (R Core Team, 2024). To assess the potential presence of publication bias in the meta-analyses, funnel plots, which display the standard error against proportion of events, were generated for all endometriosis sites, and the Egger’s tes was used to assess small study effects (Egger *et al.*, 1997). These statistical analyses were performed using Stata (StataCorp, 2015).

## Results

### Study selection

**Figure 1.**
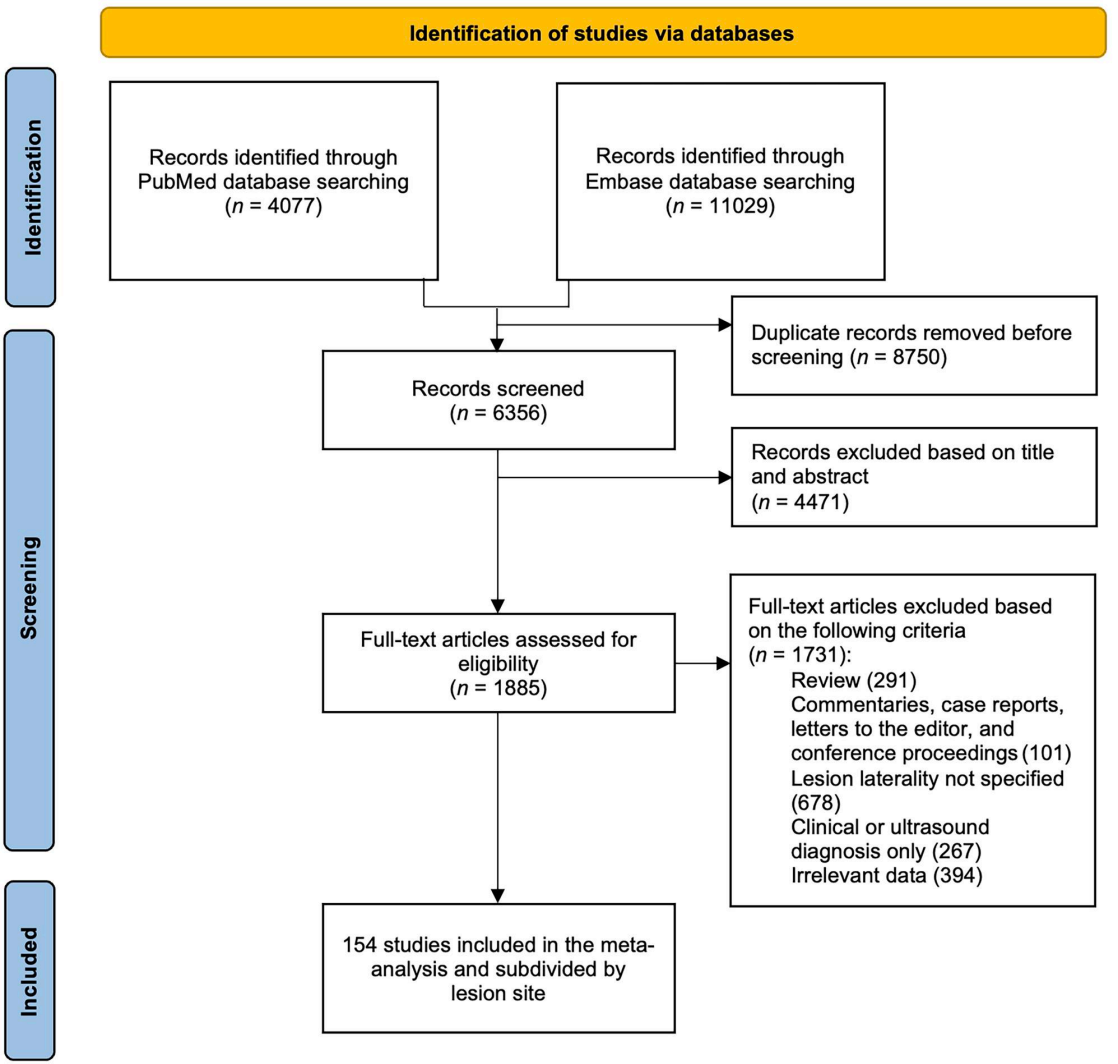
**Preferred Reporting Items for Systematic Reviews and Meta-Analyses (PRISMA) flow diagram of the literature search and study selection process for a systematic review and meta-analysis of the lateral distribution of endometriotic lesions**.

The flowchart illustrating the study screening process is shown in Fig. 1. A total of 154 reports were finally included in the quantitative synthesis. The characteristics of the selected studies are detailed in Supplementary Tables S1, S2, S3, S4, S5, and S6. Data on the laterality of lesions in patients who underwent surgery were extrapolated for the ovary (54 reports), ureter (31 reports), thorax (30 reports), uterosacral ligaments (30 reports), colon (25 reports), and inguinal region (18 reports). The sum of these articles (188) exceeds the number of studies included in the meta-analysis (154) by 34 because some articles analysed two or more endometriotic lesion types at the same time.

### Quality of evidence

Based on the ROBINS-I tool, we identified 35 studies with a low risk of bias, 46 with a moderate risk of bias, and 2 with a high risk of bias. Moreover, 71 case series were analysed using the evidence synthesis tool developed by Murad *et al.* (2018) and 14 studies (20%) received the highest rating (6), 41 (57%) received a rating of 5, 12 (17%) of 4, two (3%) received a rating of 3, and two (3%) received the lowest rating of 2. These data were collected and reported in Supplementary Tables S7 and S8.

### Meta-analysis

#### Left preponderance: ovarian, USL, ureteral, and colon endometriosis

**Figure 2.**
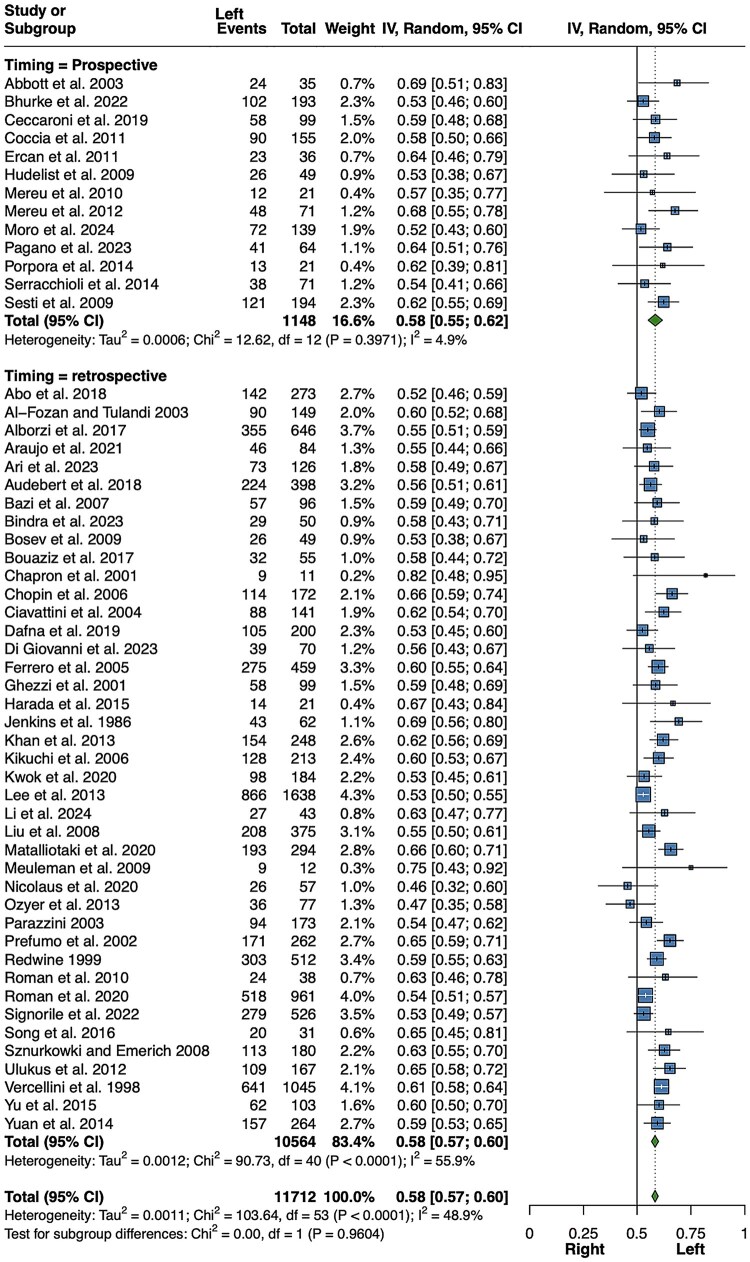
**Meta-analysis of the proportions of left-sided ovarian endometriomas (including also studies in which it was not explicitly specified whether lesions were unilateral or bilateral)**. Random, random effect model.

A meta-analysis of 54 studies included 11 712 patients with surgically removed unilateral ovarian endometriomas (Jenkins *et al.*, 1986; Vercellini *et al.*, 1998; Redwine, 1999; Chapron *et al.*, 2001; Ghezzi *et al.*, 2001; Prefumo *et al.*, 2002; Abbott *et al.*, 2003; Al-Fozan and Tulandi, 2003; Parazzini, 2003; Ciavattini *et al.*, 2004; Ferrero *et al.*, 2005; Chopin *et al.*, 2006; Kikuchi *et al.*, 2006; Bazi *et al.*, 2007; Liu *et al.*, 2008; Sznurkowski and Emerich, 2008; Bosev *et al.*, 2009; Hudelist *et al.*, 2009; Meuleman *et al.*, 2009; Sesti *et al.*, 2009; Mereu *et al.*, 2010, 2012; Roman *et al.*, 2010, 2020; Coccia *et al.*, 2011; Ercan *et al.*, 2011; Ulukus *et al.*, 2012; Khan *et al.*, 2013; Lee *et al.*, 2013; Özyer *et al.*, 2013; Porpora *et al.*, 2014; Seracchioli *et al.*, 2014; Yuan *et al.*, 2014; Harada *et al.*, 2015; Yu *et al.*, 2015; Song *et al.*, 2016; Alborzi *et al.*, 2017; Bouaziz *et al.*, 2017; Abo *et al.*, 2018; Audebert *et al.*, 2018; Ceccaroni *et al.*, 2019; Dafna *et al.*, 2019; Kwok *et al.*, 2020; Matalliotaki *et al.*, 2020; Nicolaus *et al.*, 2020; Araujo *et al.*, 2021; Bhurke *et al.*, 2022; Signorile *et al.*, 2022; Ari *et al.*, 2023; Bindra *et al.*, 2023; Di Giovanni *et al.*, 2023; Pagano *et al.*, 2023; Li *et al.*, 2024; Moro *et al.*, 2024). Of these, 6723 were located on the left side. As shown in Fig. 2, the imbalance in the proportion of left-sided lesions is statistically significant (58%; 95% CI: 57–60%, *P *< 0.001; *I*^2^ = 48.9%, *P *< 0.001). Moreover, when a subgroup analysis was performed separately for prospective and retrospective studies, the preponderance of left laterality remained statistically significant (both, *P *< 0.001), but without significant heterogeneity in the prospective design group (13 reports, *I*^2^ = 4.9%, *P *= 0.397). The results did not change when a further sensitivity analysis was conducted, considering only those studies in which it was unequivocally specified that the patient population exclusively had unilateral lesions, thus excluding 3032 patients with bilateral cysts (Supplementary Fig. S1).

**Figure 3.**
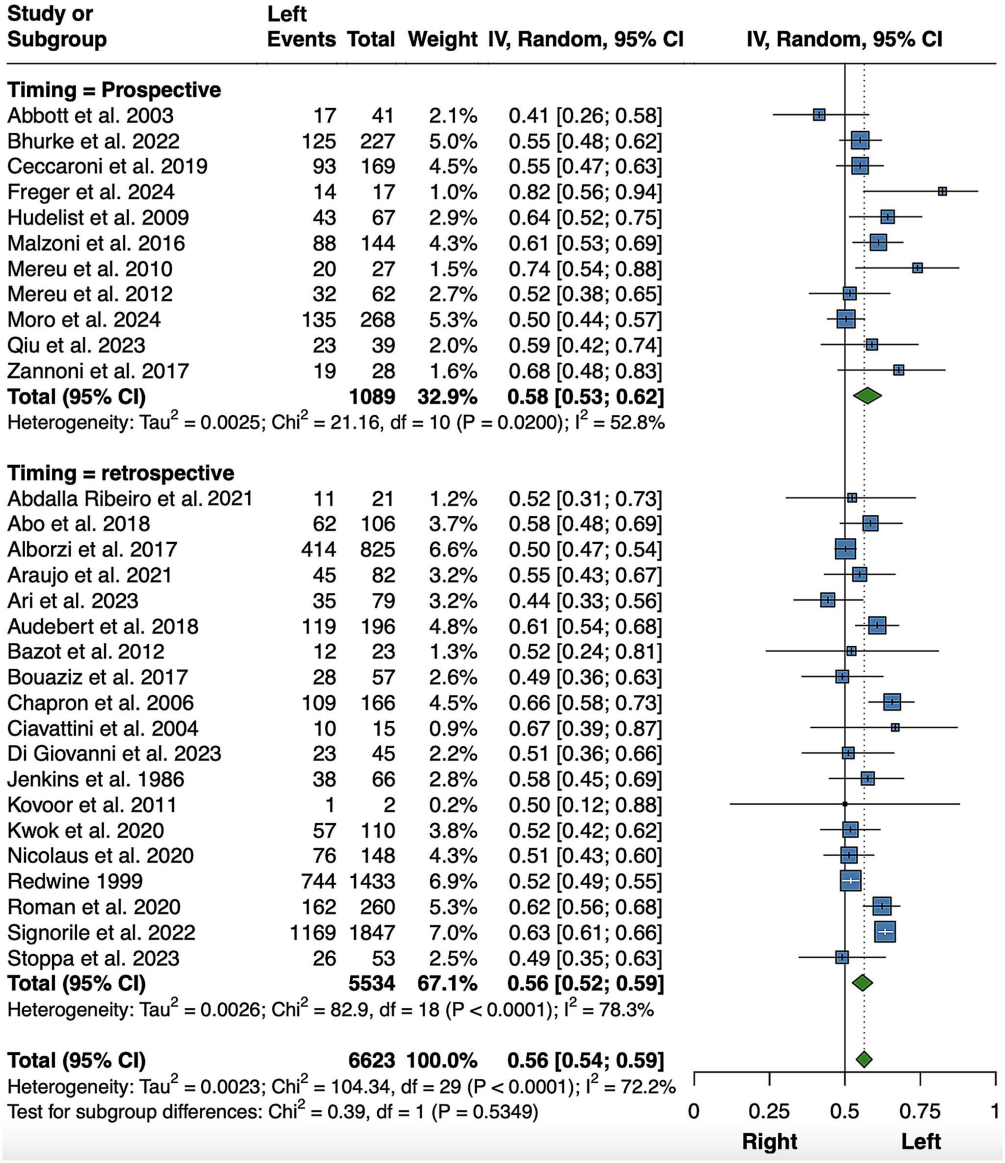
**Meta-analysis of the proportions of left-sided uterosacral ligament lesions (including also studies in which it was not explicitly specified whether lesions were unilateral or bilateral)**. Random, random effect model.

Similarly, endometriotic lesions of the uterosacral ligaments also showed a statistically significant asymmetry, with 3750 left-sided lesions out of a total of 6623 unilateral lesions (56%; 95% CI 54–59%, *P *< 0.001) (Fig. 3). These data were retrieved from 30 reports (Jenkins *et al.*, 1986; Redwine, 1999; Abbott *et al.*, 2003; Ciavattini *et al.*, 2004; Chapron *et al.*, 2006; Hudelist *et al.*, 2009; Mereu *et al.*, 2010, 2012; Kovoor *et al.*, 2011; Bazot *et al.*, 2012; Malzoni *et al.*, 2016; Alborzi *et al.*, 2017; Bouaziz *et al.*, 2017; Zannoni *et al.*, 2017; Abo *et al.*, 2018; Audebert *et al.*, 2018; Ceccaroni *et al.*, 2019; Kwok *et al.*, 2020; Nicolaus *et al.*, 2020; Roman *et al.*, 2020; Abdalla Ribeiro *et al.*, 2021; Araujo *et al.*, 2021; Bhurke *et al.*, 2022; Signorile *et al.*, 2022; Ari *et al.*, 2023; Di Giovanni *et al.*, 2023; Qiu *et al.*, 2023; Stoppa *et al.*, 2023; Freger *et al.*, 2024; Moro *et al.*, 2024). A subgroup meta-analysis based on prospective versus retrospective design was performed due to the significant heterogeneity observed. The statistically significant preponderance of left lesions was confirmed in both subgroups (*P *< 0.001), with a reduction in heterogeneity among prospective studies. A first sensitivity analysis was performed after removing the study by Signorile *et al.* (2022), as three-quarters of the patients included were diagnosed based on clinical criteria alone. However, this did not substantially change the results (56%; 95% CI 53–58%, *P *< 0.001; *I*^2^=51.1%, *P *< 0.001; data not shown). A further sensitivity analysis, performed after excluding three studies (Jenkins *et al.*, 1986; Malzoni *et al.*, 2016; Bouaziz *et al.*, 2017) that only described intraoperative visualization of the USL lesion, but not surgical excision, did not change the findings (56%; 95% CI 53–59%, *P *< 0.001; *I*^2^ = 74.5%, *P *< 0.001; data not shown). Again, a sensitivity analysis carried out by including only articles with exclusively confirmed unilateral lesions produced comparable results, but with a further reduction in heterogeneity (Supplementary Fig. S2). Here, 818 patients with bilateral USL endometriosis were not included.

**Figure 4.**
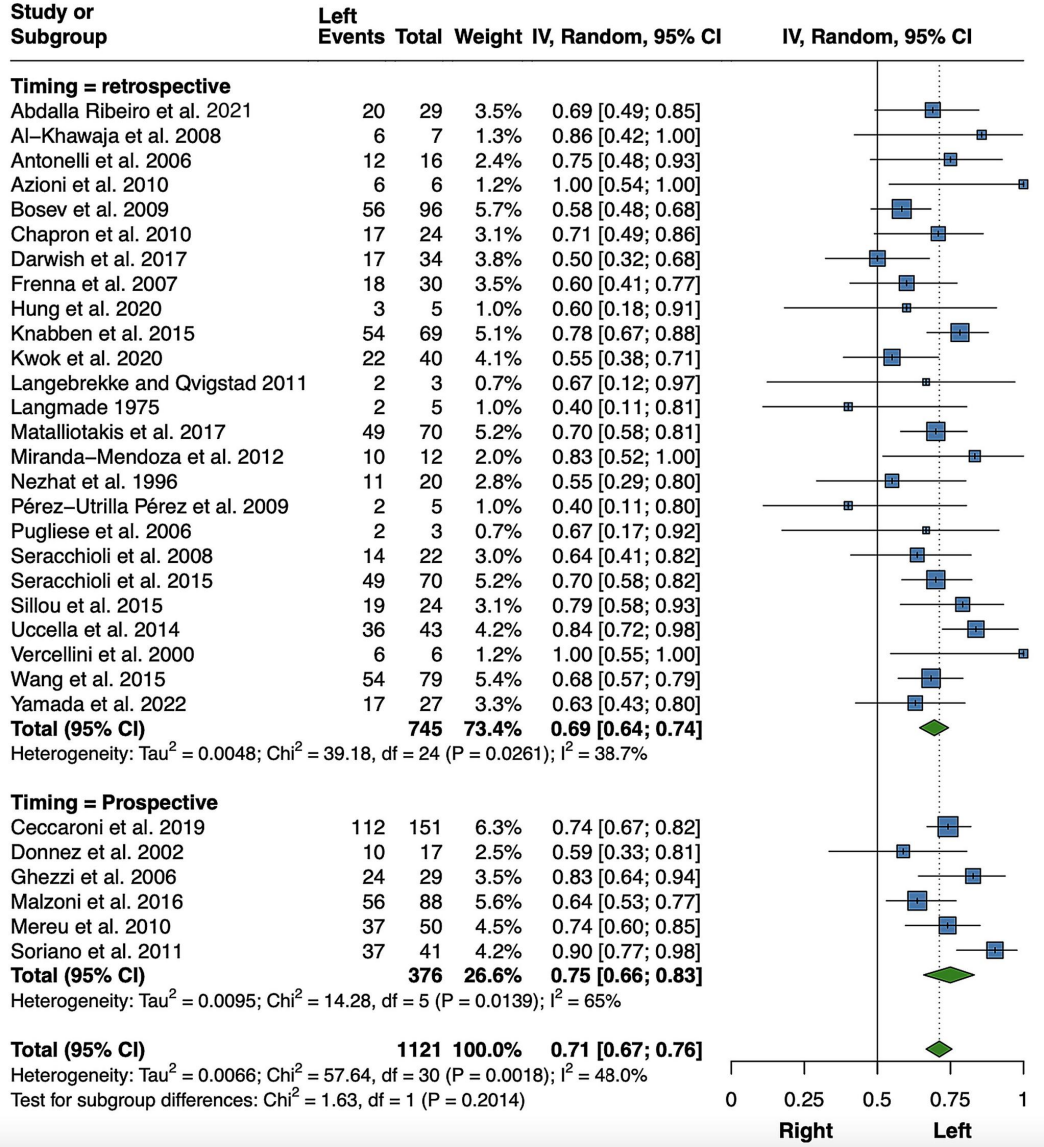
**Meta-analysis of the proportions of left-sided ureteral lesions (including also studies in which it was not explicitly specified whether lesions were unilateral or bilateral)**. Random, random effect model.

Ureteral lesions also exhibited a left-sided preponderance, based on data from 31 reports (Langmade, 1975; Nezhat *et al.*, 1996; Vercellini *et al.*, 2000b; Donnez *et al.*, 2002; Antonelli *et al.*, 2006; Ghezzi *et al.*, 2006; Pugliese *et al.*, 2006; Frenna *et al.*, 2007; Al-Khawaja *et al.*, 2008; Seracchioli *et al.*, 2008, 2015; Bosev *et al.*, 2009; Pérez-Utrilla Pérez *et al.*, 2009; Azioni *et al.*, 2010; Chapron *et al.*, 2010; Mereu *et al.*, 2010; Langebrekke and Qvigstad, 2011; Soriano *et al.*, 2011; Miranda-Mendoza *et al.*, 2012; Uccella *et al.*, 2014; Knabben *et al.*, 2015; Sillou *et al.*, 2015; Wang *et al.*, 2015; Malzoni *et al.*, 2016; Darwish *et al.*, 2017; Matalliotakis *et al.*, 2017; Ceccaroni *et al.*, 2019; Hung *et al.*, 2020; Kwok *et al.*, 2020; Abdalla Ribeiro *et al.*, 2021; Yamada *et al.*, 2022). As shown in Fig. 4, 780 out of 1121 endometriotic lesions were identified on the left side (71%; 95% CI 67–76%, *P *< 0.001; *I*^2^ = 48%, *P *= 0.002). As various radical and non-radical surgical techniques were reported (Supplementary Table S3), including ureteral resection with ureteroureterostomy or ureteroneocystostomy, ureterolysis, ureteral nodule excision, and nephroureterectomy, a sensitivity analysis was performed after removing studies that included only patients who underwent ureterolysis. The estimate of left laterality remained essentially unchanged, but the heterogeneity was no longer significant (Supplementary Fig. S3). The result of the meta-analysis was substantially similar (71%; 95% CI 66–75%, *P *< 0.001; *I*^2^ = 45.4%, *P *= 0.004; data not shown) when a sensitivity analysis was conducted after excluding the study by Uccella *et al.* (2014), as ureteral lesion laterality was assessed preoperatively by ultrasound in the presence of hydronephrosis. Moreover, the results did not change substantially when only studies with exclusively unilateral lesions were considered, thus excluding 155 patients with bilateral ureteral endometriosis (Supplementary Fig. S4).

**Figure 5.**
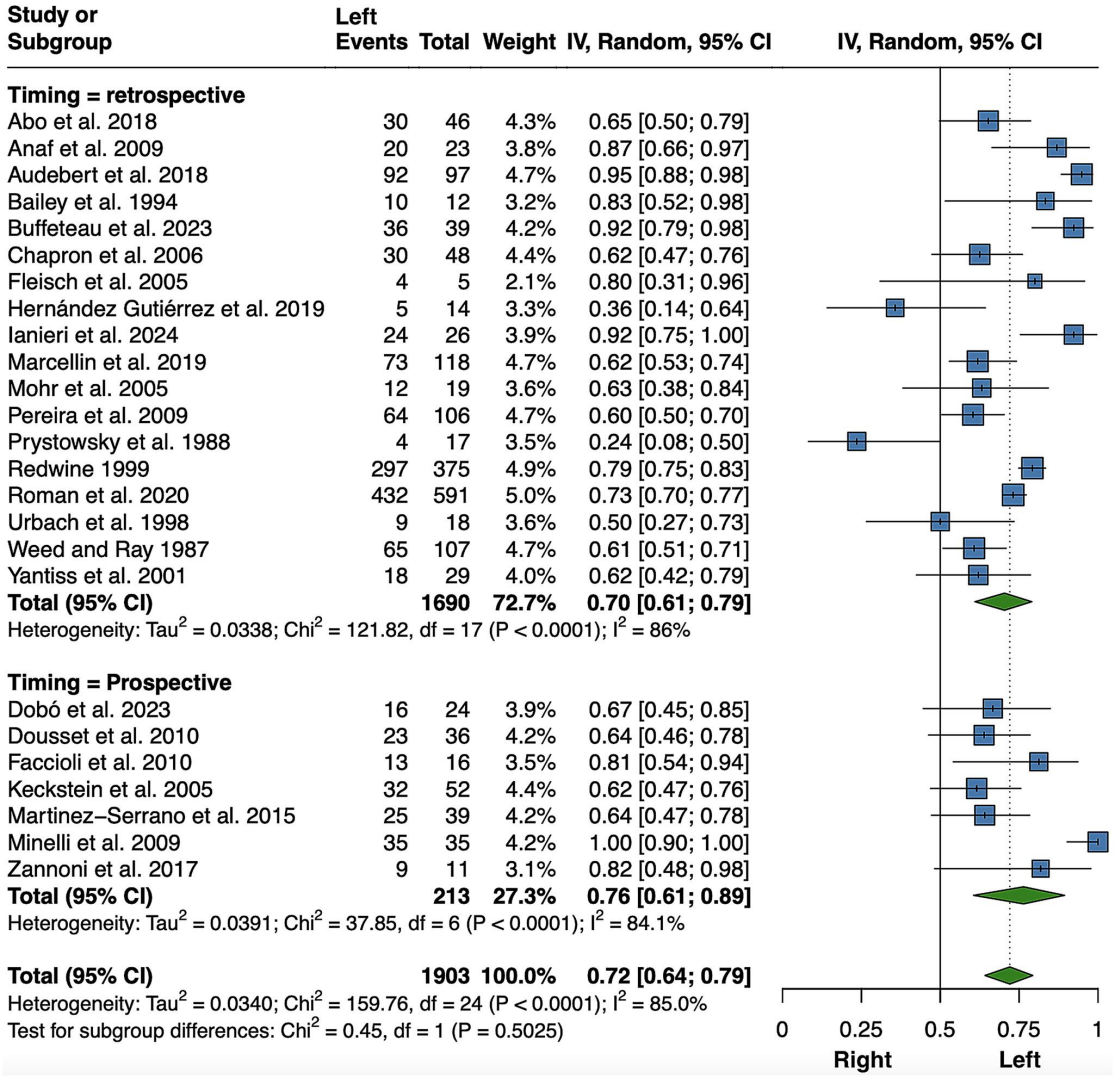
**Meta-analysis of the proportions of left-sided bowel lesions (including also studies in which it was not explicitly specified whether lesions were unilateral or bilateral)**. Random, random effect model.

A total of 1378 out of the 1903 patients included in the 25 selected articles on bowel endometriosis (Weed and Ray, 1987; Prystowsky *et al.*, 1988; Bailey *et al.*, 1994; Urbach *et al.*, 1998; Redwine, 1999; Yantiss *et al.*, 2001; Fleisch *et al.*, 2005; Keckstein and Wiesinger, 2005; Mohr *et al.*, 2005; Chapron *et al.*, 2006; Anaf *et al.*, 2009; Minelli *et al.*, 2009; Pereira *et al.*, 2009; Dousset *et al.*, 2010; Faccioli *et al.*, 2010; Martínez-Serrano *et al.*, 2015; Zannoni *et al.*, 2017; Abo *et al.*, 2018; Audebert *et al.*, 2018; Hernández Gutiérrez *et al.*, 2019; Marcellin *et al.*, 2019; Roman *et al.*, 2020; Buffeteau *et al.*, 2023; Dobó *et al.*, 2023; Ianieri *et al.*, 2024) had colon lesions on the left side (Fig. 5), with a marked left-sided preponderance (72%; 95% CI 64–79%, *P *< 0.001; *I*^2^ = 85%, *P *< 0.001). Initially, only articles evaluating major surgery for bowel endometriosis were included. The laterality of resected bowel lesions was extrapolated from some reports, excluding cases treated by shaving or disc excision (Supplementary Table S4). However, as this was not always feasible, a sensitivity analysis was performed including only studies of patients who underwent segmental bowel resection, and the result was similar (Supplementary Fig. S5). The results did not change substantially when only studies describing patients with exclusively unilateral lesions were considered, as only 44 patients with clearly described bilateral colonic lesions were excluded. However, the heterogeneity was markedly decreased and no longer significant in the prospective group (Supplementary Fig. S6).

Funnel plots were generated for all four endometriosis sites to assess the potential presence of publication bias in the meta-analyses (Supplementary Figs S7, S8, S9, and S10). The plots show the standard error against the proportion of events, with a symmetrical distribution of studies around the pooled estimate. Egger’s test for small study effects showed no significant publication bias for the meta-analyses of uterosacral ligaments, ureter, and bowel endometriosis (*P *= 0.964; *P *= 0.992; and *P *= 0.377, respectively; Supplementary Figs S8, S9, and S10).

Conversely, the funnel plot for ovarian endometriomas shows an asymmetric distribution of studies around the pooled estimate, suggesting publication bias (Egger test, *P *= 0.012; Supplementary Fig. S7). To better understand the impact of this bias on the estimate of ovarian lesion laterality, we performed some in-depth analyses. We conducted a subgroup analysis dividing small studies (<50 participants) from large studies (≥50 participants) and calculated Egger’s test excluding small studies. These two analyses show that Egger’s test on the subgroup of large studies is not significant (*P *= 0.068; data not shown) and that the overall meta-analysis estimate is higher for small studies (left laterality = 62.0%; data not shown), and lower and comparable when considering only large studies (left laterality = 58.3%; data not shown) or all studies combined (left laterality = 58.7%).

#### Right preponderance: thoracic and inguinal endometriosis

For these two sites, the populations of women with unilateral and bilateral lesions were always clearly distinguished in all the studies considered.

**Figure 6.**
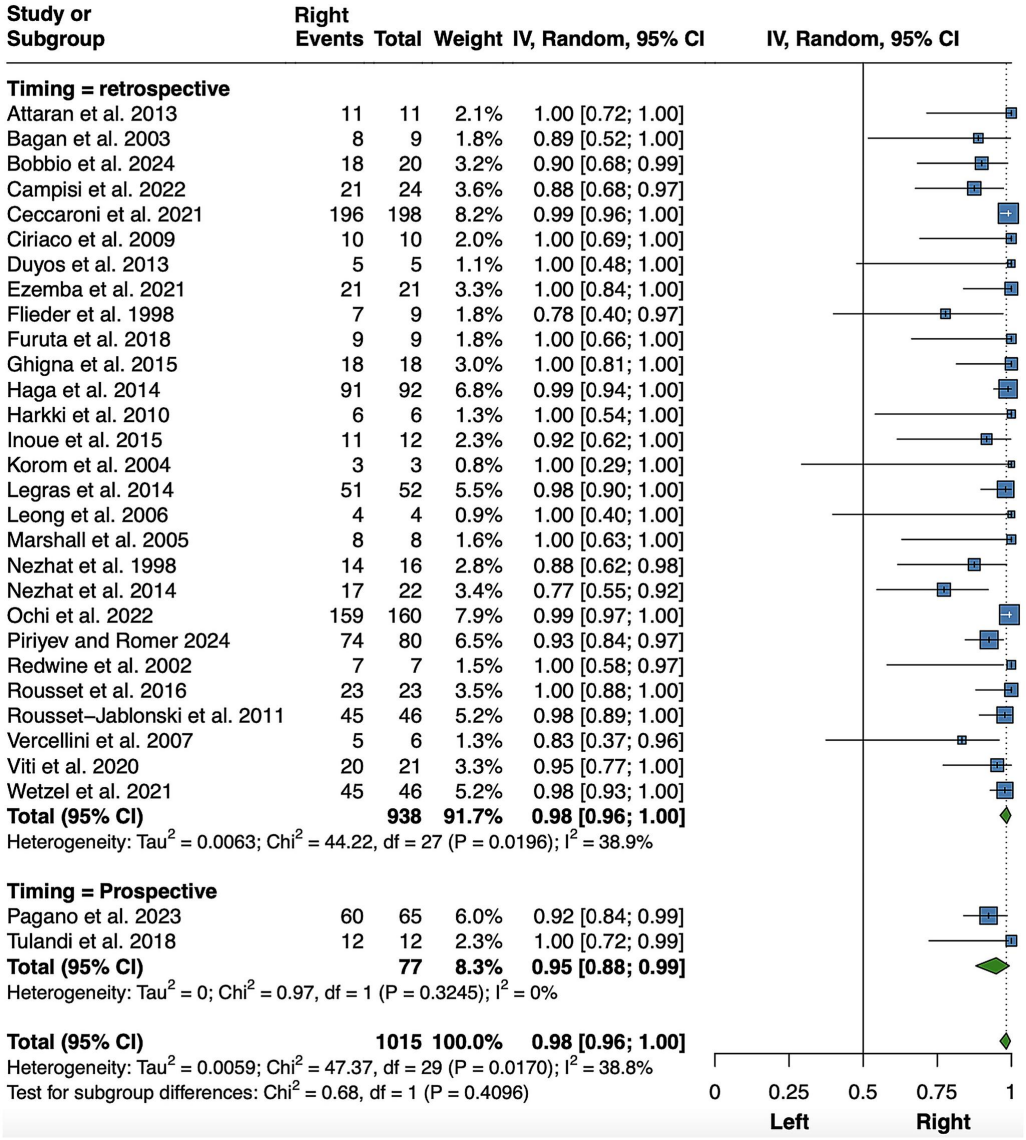
**Meta-analysis of the proportions of right-sided thoracic lesions among unilateral lesions.** Random, random effect model.

For thoracic endometriosis, 30 studies were identified that focused on diaphragmatic, pleural, and pulmonary lesions, either alone or in combination (Flieder *et al.*, 1998; Nezhat *et al.*, 1998, 2014; Redwine, 2002; Bagan *et al.*, 2003; Korom *et al.*, 2004; Marshall *et al.*, 2005; Leong *et al.*, 2006; Vercellini *et al.*, 2007; Ciriaco *et al.*, 2009; Härkki *et al.*, 2010; Rousset-Jablonski *et al.*, 2011; Attaran *et al.*, 2013; Duyos *et al.*, 2014; Haga *et al.*, 2014; Legras *et al.*, 2014; Ghigna *et al.*, 2015; Inoue *et al.*, 2015; Rousset *et al.*, 2016; Furuta *et al.*, 2018; Tulandi *et al.*, 2018; Viti *et al.*, 2020; Ceccaroni *et al.*, 2021; Ezemba *et al.*, 2021; Wetzel *et al.*, 2021; Campisi *et al.*, 2022; Ochi *et al.*, 2022; Pagano *et al.*, 2023; Bobbio *et al.*, 2024; Piriyev and Römer, 2024). Of 1015 patients, 979 had right-sided lesions, indicating that almost all unilateral thoracic lesions were located on the right side (98%; 95% CI 96–100%; *P *< 0.001; *I*^2^ = 38.8%, *P *= 0.017) (Fig. 6). A subgroup analysis was conducted with studies categorized as retrospective or prospective. No heterogeneity was identified in the prospective group. To avoid potential bias due to the surgical difficulty and feasibility of identifying diaphragmatic lesions, a sensitivity analysis was also conducted, including patients with pleural endometriotic lesions only or studies explicitly stating that the entire bilateral hemidiaphragms were examined. The result was almost identical, but without heterogeneity (Supplementary Fig. S11). A further 66 patients with bilateral thoracic lesions were not considered.

**Figure 7.**
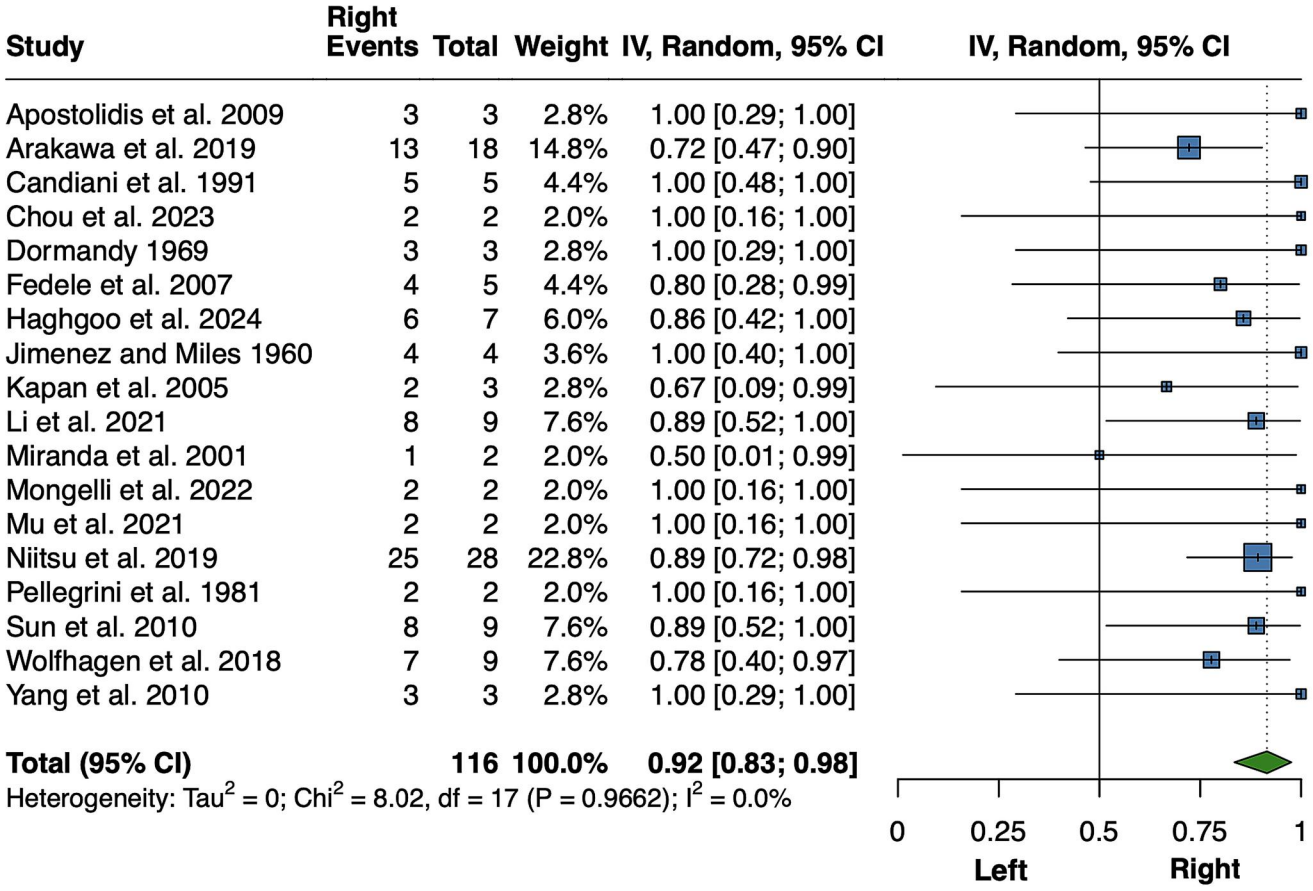
**Meta-analysis of the proportions of right-sided inguinal lesions among unilateral lesions.** Random, random effect model.

Although rare, inguinal endometriosis has been described in several case series. In our meta-analysis, shown in Fig. 7, data were collected from 18 studies comprising 116 patients (Dormandy, 1956; Jimenez and Miles, 1960; Pellegrini *et al.*, 1981; Candiani *et al.*, 1991; Miranda *et al.*, 2001; Kapan *et al.*, 2005; Fedele *et al.*, 2007; Apostolidis *et al.*, 2009; Sun *et al.*, 2010; Yang *et al.*, 2010; Wolfhagen *et al.*, 2018; Arakawa *et al.*, 2019; Niitsu *et al.*, 2019; Li *et al.*, 2021; Mu *et al.*, 2021; Mongelli *et al.*, 2022; Chou *et al.*, 2023; Haghgoo *et al.*, 2024). A total of 100 patients had inguinal lesions on the right side (92%; 95% CI 83–98%, *P *< 0.001; *I*^2^=0%, *P *= 0.966). A sensitivity analysis was performed after removing the studies by Arakawa *et al.* (2019) and Haghgoo *et al.* (2024), as some lesions were assessed by magnetic resonance imaging or ultrasound rather than surgical excision. However, the results remained essentially unchanged (94%; 95% CI 86–100%, *P *< 0.001; *I*^2^ = 0%, *P *= 0.983; data not shown). Only five excluded patients had bilateral inguinal lesions.

The funnel plots for thoracic and inguinal endometriosis did not suggest a publication bias by visual inspection (Supplementary Figs S12 and S13). No significant small study effect was detected at Egger’s test (*P *= 0.134 and *P *= 0.272, respectively).

## Discussion

### Main findings

After pooling a large amount of data, we observed a consistent and statistically significant asymmetry in the lateral distribution of potentially bilateral endometriotic lesions (left preponderance: ovary, 58%; USL, 56%; ureter, 71%; colon, 72%; and right preponderance: thorax, 98%; inguinal, 92%).

In addition, the asymmetry was observed on either the right or left side, depending on the type of lesion considered, thus supporting the hypothesis that endometriosis is an asymmetrical disease. The side preponderance appears to be lesion-specific and may be determined by physical (gravity), anatomical (organs and structures that channel refluxed blood and endometrium into protected niches), and physiological (clockwise peritoneal fluid flow, colonic peristalsis, and the rhythmic up and down movements of the diaphragm, a ‘thoracoabdominal pump’ that causes rapid inversions of hydrostatic pressures) factors (Vercellini *et al.*, 2007; Bricou *et al.*, 2008).

The rectosigmoid may shelter endometrial fragments refluxed through the left tube, creating local anatomical niches. The left hemipelvis would thus be protected from the peritoneal fluid flow and the intense ileal peristalsis, factors that should generally interfere with cell attachment. This could favour the implantation of endometrial cells on the surface of the left structures (Vercellini *et al.*, 1998, 2000b, 2004; Al-Fozan and Tulandi, 2003; Parazzini, 2003; Chapron *et al.*, 2006; Scioscia *et al.*, 2011; Audebert *et al.*, 2018; Kwok *et al.*, 2020; Araujo *et al.*, 2021; Pagano *et al.*, 2023). The peritoneum is laid like a sheet over strings formed by the uterosacral ligaments. This creates a deep central retro-uterine pouch (pouch of Douglas) which is covered by the anterior rectal wall. Rectovaginal endometriosis develops in this protected anatomical recess. Two smaller pouches are formed on the lateral aspects of both uterosacral ligaments. However, the sigmoid mainly protects the left uterosacral pouch, as the cecum lies more cranially. This may explain the lateral preponderance of left uterosacral endometriotic nodules (Chapron *et al.*, 2001) and ureteral endometriosis. Indeed, obstructive uropathy very often occurs as a secondary manifestation of extensive fibrosis of the lateral parametrium.

The right preponderance of inguinal endometriosis is likely also explained by the relatively cranial position of the cecum, which does not prevent endometrial fragments from being channelled through the ipsilateral internal inguinal ring (Candiani *et al.*, 1991). The right preponderance of the right diaphragmatic and pleural endometriotic lesions appears to be caused by endometrial fragments being transported by the clockwise peritoneal fluid flow along the right paracolic gutter and the right hemidiaphragm, only to be blocked by the anatomical barrier created by the falciform ligament (Vercellini *et al.*, 2007; Bricou *et al.*, 2008). The anatomical recess formed by the posterior hepatic edge, the abdominal diaphragmatic surface, and the falciform ligament favours local implantation of the endometrial fragments. During inspiration, the diaphragm descends, reducing the intrathoracic negative pressure and increasing the intra-abdominal pressure. This thoracoabdominal pump may facilitate the upward migration of endometrial cells that would be ‘sucked in’ through small diaphragm defects or cribriform fenestrations and remain trapped under the right basal pleural layer (Bricou *et al.*, 2008).

Thus, the findings of the present overview confirm the possible existence of two main asymmetric compartments where transtubally refluxed endometrial fragments may more easily implant due to the creation of anatomical recesses and protection from the peritoneal fluid flow, i.e. a left hemipelvic endometriosis compartment, including the left ovary, uterosacral ligament, ureter and sigmoid colon, and a right hypochondrium endometriosis compartment, including the right diaphragm and pleura (Vercellini *et al.*, 2007; Bricou *et al.*, 2008; Rousset-Jablonski *et al.*, 2011; Pagano *et al.*, 2023; Dridi *et al.*, 2025).

### Strengths and limitations

This comprehensive meta-analysis included a total of 20 718 patients of different nationalities and ethnicities from 154 studies conducted over almost four decades in 29 countries on five continents. The large amount of data and the consistency of the results observed across different types of endometriosis support the validity of the study hypothesis and allow generalization of the results. The PRISMA guidelines were carefully followed, and the literature searches and data extraction carried out in the different participating centres were systematically double-checked by two authors (V.B. and C.P.) from the coordinating centre.

Selection, ascertainment, and reporting bias cannot be completely ruled out, particularly for lesions that are difficult to identify, such as endometriotic diaphragmatic nodules/cysts, which are usually hidden behind the right hepatic lobe and can be visualized at laparoscopy by pushing down the liver parenchyma with a blunt probe (Nezhat *et al.*, 1998). Regarding bowel and USL lesions, substantial heterogeneity could be explained by variability in surgical approaches, both across studies and sometimes even within the same cohort. For uterosacral lesions, heterogeneity may also reflect differences in the operator’s awareness and accuracy in describing intraoperative findings. Although restricting the analysis to patients with pathologically proven disease reduced bias related to uncertain diagnoses, heterogeneity in the USL meta-analysis remained high, even when limiting the analysis to studies involving major surgery. For bowel endometriosis, we hypothesized that an additional source of variability might be the difficulty in determining whether multiple resections indicated bilateral disease or multiple lesions on the same side. Heterogeneity persisted even when analyses were restricted to studies that explicitly distinguished unilateral from bilateral cases. Moreover, most of these studies were retrospective (18 out of 25) and based on small cohorts, factors that may have contributed to variability. Overall, despite several subgroup and sensitivity analyses, residual heterogeneity could not be eliminated, indicating that pooled estimates for these sites should be interpreted with caution.

In addition, a publication bias cannot be ruled out. Consistent with this, Egger’s test indicated a potential small study effect in the meta-analysis of ovarian endometriomas (*P *= 0.012), suggesting a cautious interpretation of the pooled estimate. Indeed, this is the most common site of endometriotic lesions, and many articles have specifically aimed to assess asymmetry at this site. In contrast, for other anatomical locations, laterality is often reported incidentally as part of other study endpoints. However, our additional analyses showed that the bias detected was related to small studies (since it was no longer significant when only larger studies were considered) and that, whether small studies are included or excluded, the overall estimate remains substantially comparable. Therefore, we believe that the effect of publication bias on the pooled estimate is negligible.

A further limitation of this review is that 126 out of the 154 included studies were retrospective, and the vast majority of the considered cohorts were small (<50 participants). This may exert a detrimental effect on the strength of our findings and may also increase the risk of publication bias.

We do not believe that the exclusion of bilateral lesions from most of the meta-analyses is a limitation of our review. Estimating laterality while considering bilateral lesions would, in fact, imply adding right (or left) lesions to both the numerator and the denominator, which is both statistically inappropriate and redundant. In addition, the main meta-analyses included studies in which unilateral and bilateral lesions may have been mixed, as this distinction was not always reported. However, in the subset of patients analysed in the sensitivity analyses, where unilateral and bilateral lesions were explicitly distinguished, the proportion of patients with bilateral lesions was a minority, i.e. 26% (3032/11 811) for ovarian endometrioma, 42% (818/1921) for USL endometriosis, 14% (155/1089) for ureteral endometriosis, 15% (44/291) for bowel endometriosis, 6% (66/1081) for thoracic endometriosis, and 4% (5/121) for inguinal endometriosis.

It is important to note that the asymmetry in the lateral distribution of the lesions was observed on both the left and the right side. This is pathogenically relevant because it seems unclear how embryological or biological mechanisms could plausibly explain the opposite lateral asymmetry of different endometriotic lesions (i.e. ovarian, intestinal and ureteral endometriosis on the left side, and inguinal, diaphragmatic, and pleural endometriosis on the right side).

Finally, our findings are consistent with those regarding asymmetry in the development of other lesion types not considered in this review, such as sciatic nerve endometriosis (Vercellini *et al.*, 2003) and clear-cell (Vercellini *et al.*, 2000c) and endometrioid ovarian cancer (Vercellini *et al.*, 2000c; McMullan *et al.*, 2024), i.e. gonadal malignancies presumably originating from ectopic endometrium.

### Comparison with existing literature

An asymmetrical lateral distribution has been reported for several types of endometriotic lesions (Chapron *et al.*, 2006; Legras *et al.*, 2014; Knabben *et al.*, 2015; Matalliotakis *et al.*, 2017; Audebert *et al.*, 2018; Haghgoo *et al.*, 2024). However, Guo *et al.* (2008), after reviewing the relevant reports published up to October 2006, observed that the excess of left-sided over right-sided cases decreased from 78% to 5% as the sample size of the studies increased. According to the authors, small studies with a higher proportion of unilateral lesions and a higher asymmetry in the lateral lesion distribution cannot be used to demonstrate the validity of the retrograde menstruation theory because the estimates are imprecise and random fluctuations of data are likely. Conversely, larger studies with a higher proportion of bilateral lesions would support the metaplasia theory (Guo *et al.*, 2008).

In our review, the heterogeneity of results for some lesion types was no longer statistically significant and/or markedly reduced when only prospective studies, which are likely to report higher quality evidence than retrospective studies, were considered. For example, for ovarian endometriomas, the *I^2^* was 4.9% in prospective studies and 48.9% in retrospective studies. The corresponding percentages for uterosacral ligament lesions were 52.8% and 72.2%. The same was true when sensitivity analyses were performed. Therefore, the inclusion of the many studies published after 2006 significantly reduced the extreme quantitative inconsistency found by Guo *et al.* (2008).

Chapron *et al.* (2001) also observed an asymmetry in the lateral distribution of deep endometriosis infiltrating the uterosacral ligaments. Nonetheless, they proposed a different pathogenic interpretation to retrograde menstruation involving implantation into anatomical niches. Since ovulation appears to occur more frequently in the right ovary, a natural progesterone exposure gradient would be created, which would act as a protective factor against endometriosis development in the right hemipelvis (Chapron *et al.*, 2001). However, this would not explain the opposite asymmetry in the distribution of endometriotic lesions in the upper abdomen and thorax.

### Implications for research and clinical practice

The results of our meta-analysis may already have clinical implications, as they support the pathogenic role of retrograde menstruation in the establishment of endometriosis and add to the recent growing body of evidence in favour of such a theory (Konrad *et al.*, 2019; Bulun, 2022; Salmeri *et al.*, 2024; Vercellini *et al.*, 2024d, e, 2025b; Viganò *et al.*, 2024). This aetiological background should be integrated into the overall clinical scenario of the endometriotic disease, which seems to be characterized by: (i) early onset in the postmenarcheal years (Hirsch *et al.*, 2020; Lu *et al.*, 2023; Vercellini *et al.*, 2024b, 2025a; Oliveira *et al.*, 2025); (ii) prolonged diagnostic delay (De Corte *et al.*, 2025); and (iii) a tendency to progress to complicated forms associated with infertility and severe symptoms, with a need for demanding surgery (Agarwal *et al.*, 2019; Ding *et al.*, 2020; Coccia *et al.*, 2022; Knez *et al.*, 2023; Bandini *et al.*, 2024). If retrograde menstruation is the trigger for the whole chain of events, suppression of ovulatory menses could be considered in adolescents and young women who complain of severe and disabling menstrual symptoms and do not respond to non-steroidal anti-inflammatory drugs (Martire *et al.*, 2023; Chapron *et al.*, 2024; Vercellini *et al.*, 2024a,c), as secondary prevention could modify the course of the disease.

## Conclusion

The results of this comprehensive systematic review and meta-analysis confirm the asymmetrical lateral distribution of endometriotic lesions in bilateral organs and structures, increase the precision of percentage estimates, and substantially reduce the previously observed quantitative heterogeneity. The difference between the two sides was statistically significant for all the different types of lesions considered. In addition, the lateral preponderance was observed on either the right or the left side, depending on the specific lesions considered. In particular, pelvic endometriotic lesions (ovaries, uterosacral ligaments, bowel) were significantly more frequent on the left side, whereas thoracic and inguinal lesions exhibited a right-side predominance. For each type of lesion, the lateral asymmetry could be explained by physical, anatomical, and dynamic/functional factors that determine the abdominal distribution and implantation of transtubally refluxed endometrial fragments. Because the coelomic metaplasia and the embryonic cell remnant theories do not appear to be compatible with the above anatomical pattern, the *in situ* development of the most commonly observed endometriotic lesions seems unlikely.

## Supplementary Material

hoaf064_Supplementary_Data

## Data Availability

The data included in this article were extracted from the published original articles. No new data were generated in support of this research.
