# Supplementary material for: Lateral distribution of endometriotic lesions: the anatomical recesses hypothesis. A systematic review and meta-analysis
Source: Hum Reprod Open. 2025 Oct 24;2026(1):hoaf064. doi: 10.1093/hropen/hoaf064 (PMC12816922; doi:10.1093/hropen/hoaf064)
Supplement: hoaf064_Supplementary_Data [file hoaf064_supplementary_data.zip › Supplementary Figures S1-13.pdf]

Study or Subgroup	Left Events	Total	Weight	IV, Random, 95% CI
**Timing = Prospective**				
Abbott et al. 2003	24	35	0.9%	0.69 [0.51; 0.83]
Coccia et al. 2011	90	155	2.6%	0.58 [0.50; 0.66]
Ercan et al. 2011	23	36	0.9%	0.64 [0.46; 0.78]
Hudelist et al. 2009	26	49	1.1%	0.53 [0.38; 0.67]
Mereu et al. 2010	12	21	0.5%	0.57 [0.35; 0.77]
Mereu et al. 2012	48	71	1.5%	0.68 [0.55; 0.78]
Pagano et al. 2023	41	64	1.4%	0.64 [0.51; 0.76]
Porpora et al. 2014	13	21	0.5%	0.62 [0.39; 0.81]
Serracchioli et al. 2014	38	71	1.5%	0.54 [0.41; 0.66]
Sesti et al. 2009	121	194	3.0%	0.62 [0.55; 0.69]
**Total (95% CI)**		**717**	**14.0%**	**0.61 [0.57; 0.65]**
Heterogeneity: $Tau^2 = 0$; $Chi^2 = 6.16$, df = 9 (P = 0.7238); $I^2 = 0\%$				
**Timing = retrospective**				
Al–Fozan and Tulandi 2003	90	149	2.5%	0.60 [0.52; 0.70]
Alborzi et al. 2017	355	646	4.9%	0.55 [0.51; 0.59]
Araujo et al. 2021	46	84	1.7%	0.55 [0.44; 0.66]
Ari et al. 2023	73	126	2.3%	0.58 [0.49; 0.67]
Audebert et al. 2018	224	398	4.1%	0.56 [0.51; 0.61]
Bazi et al. 2007	57	96	1.9%	0.59 [0.49; 0.70]
Bindra et al. 2023	29	50	1.2%	0.58 [0.43; 0.71]
Bosev et al. 2009	26	49	1.1%	0.53 [0.38; 0.67]
Bouaziz et al. 2017	32	55	1.2%	0.58 [0.44; 0.72]
Chapron et al. 2001	9	11	0.3%	0.82 [0.48; 0.95]
Chopin et al. 2006	114	172	2.8%	0.66 [0.59; 0.74]
Ciavattini et al. 2004	88	141	2.5%	0.62 [0.54; 0.70]
Dafna et al. 2019	105	200	3.0%	0.53 [0.45; 0.60]
Ferrero et al. 2005	275	459	4.4%	0.60 [0.55; 0.64]
Ghezzi et al. 2001	58	99	1.9%	0.59 [0.48; 0.68]
Harada et al. 2015	14	21	0.5%	0.67 [0.43; 0.85]
Jenkins et al. 1986	43	62	1.4%	0.69 [0.56; 0.80]
Khan et al. 2013	154	248	3.4%	0.62 [0.56; 0.68]
Kikuchi et al. 2006	128	213	3.1%	0.60 [0.53; 0.67]
Kwok et al. 2020	98	184	2.9%	0.53 [0.46; 0.61]
Lee et al. 2013	866	1638	5.8%	0.53 [0.50; 0.56]
Li et al. 2024	27	43	1.0%	0.63 [0.47; 0.77]
Liu et al. 2008	208	375	4.1%	0.55 [0.50; 0.61]
Matalliotaki et al. 2020	193	294	3.7%	0.66 [0.60; 0.71]
Meuleman et al. 2009	9	12	0.3%	0.75 [0.43; 0.93]
Ozyer et al. 2013	36	77	1.6%	0.47 [0.35; 0.58]
Parazzini 2003	94	173	2.8%	0.54 [0.47; 0.62]
Prefumo et al. 2002	171	262	3.5%	0.65 [0.59; 0.71]
Roman et al. 2010	24	38	0.9%	0.63 [0.46; 0.78]
Song et al. 2016	20	31	0.8%	0.65 [0.45; 0.81]
Sznurkowki and Emerich 2008	113	180	2.8%	0.63 [0.55; 0.70]
Ulukus et al. 2012	109	167	2.7%	0.65 [0.58; 0.72]
Vercellini et al. 1998	641	1045	5.4%	0.61 [0.58; 0.64]
Yuan et al. 2014	157	264	3.5%	0.59 [0.53; 0.65]
**Total (95% CI)**		**8062**	**86.0%**	**0.59 [0.57; 0.61]**
Heterogeneity: $Tau^2 = 0.0012$; $Chi^2 = 72.74$, df = 33 (P < 0.0001); $I^2 = 54.6\%$				
**Total (95% CI)**		**8779**	**100.0%**	**0.59 [0.58; 0.61]**
Heterogeneity: $Tau^2 = 0.0010$; $Chi^2 = 80.73$, df = 43 (P = 0.0004); $I^2 = 46.7\%$				
Test for subgroup differences: $Chi^2 = 0.59$, df = 1 (P = 0.4415)				

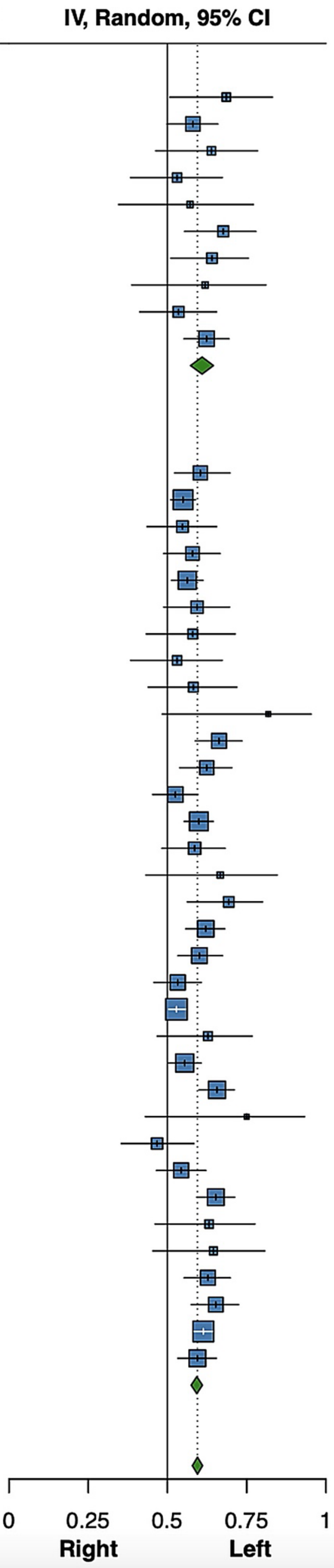

Supplementary Figure S1: Sensitivity analysis: meta-analysis of the proportions of left-sided ovarian endometriomas among explicitly specified unilateral lesions.

Abbreviations: Random, random effect model; CI, Confidence Interval.

Study or Subgroup	Left Events	Total	Weight	IV, Random, 95% CI
**Timing = Prospective**				
Abbott et al. 2003	17	41	4.8%	0.41 [0.26; 0.58]
Freger et al. 2024	14	17	2.4%	0.82 [0.57; 0.96]
Hudelist et al. 2009	43	67	6.6%	0.64 [0.52; 0.75]
Malzoni et al. 2016	88	144	10.0%	0.61 [0.53; 0.69]
Mereu et al. 2010	20	27	3.5%	0.74 [0.54; 0.88]
**Total (95% CI)**		**296**	**27.3%**	**0.63 [0.51; 0.75]**
Heterogeneity: $Tau^2 = 0.0131$; $Chi^2 = 11.75$, df = 4 (P = 0.0193); $I^2 = 65.9\%$				
**Timing = retrospective**				
Abdalla Ribeiro et al. 2021	11	21	2.8%	0.52 [0.31; 0.73]
Araujo et al. 2021	45	82	7.5%	0.55 [0.43; 0.67]
Audebert et al. 2018	119	196	11.3%	0.61 [0.54; 0.68]
Bouaziz et al. 2017	28	57	6.0%	0.49 [0.35; 0.63]
Chapron et al. 2006	109	166	10.6%	0.66 [0.58; 0.73]
Ciavattini et al. 2004	10	15	2.1%	0.67 [0.35; 0.97]
Di Giovanni et al. 2023	23	45	5.1%	0.51 [0.36; 0.66]
Kovoor et al. 2011	1	2	0.4%	0.50 [0.12; 0.88]
Kwok et al. 2020	57	110	8.8%	0.52 [0.42; 0.62]
Roman et al. 2020	162	260	12.4%	0.62 [0.56; 0.68]
Stoppa et al. 2023	26	53	5.7%	0.49 [0.35; 0.63]
**Total (95% CI)**		**1007**	**72.7%**	**0.58 [0.53; 0.62]**
Heterogeneity: $Tau^2 = 0.0018$; $Chi^2 = 13.63$, df = 10 (P = 0.1908); $I^2 = 26.6\%$				
**Total (95% CI)**		**1303**	**100.0%**	**0.59 [0.55; 0.63]**
Heterogeneity: $Tau^2 = 0.0022$; $Chi^2 = 26.13$, df = 15 (P = 0.0366); $I^2 = 42.6\%$				
Test for subgroup differences: $Chi^2 = 0.77$, df = 1 (P = 0.3796)				

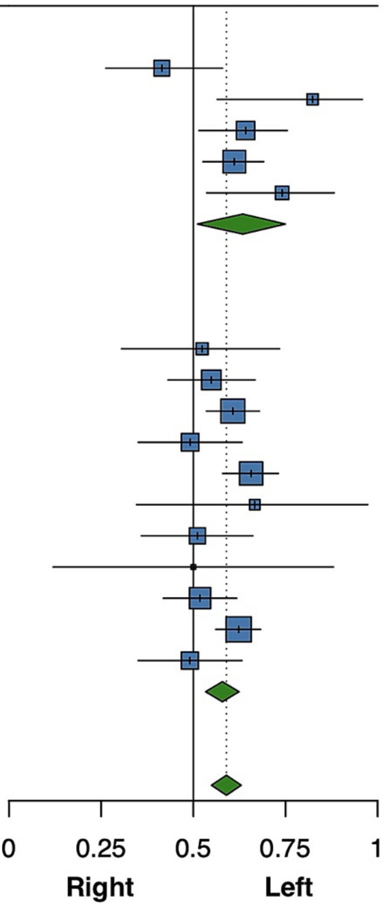

Supplementary Figure S2: Sensitivity analysis: meta-analysis of the proportions of left-sided uterosacral ligament lesions among explicitly specified unilateral lesions.
Abbreviations: Random, random effect model; CI, Confidence Interval.

Study	Left Events	Total	Weight	IV, Random, 95% CI
Abdalla Ribeiro et al. 2020	20	29	5.7%	0.69 [0.49; 0.85]
Al–Khawaja et al. 2008	6	7	1.5%	0.86 [0.42; 1.00]
Antonelli et al. 2006	12	16	3.2%	0.75 [0.48; 0.93]
Azioni et al. 2010	6	6	1.3%	1.00 [0.54; 1.00]
Ceccaroni et al. 2019	112	151	27.3%	0.74 [0.66; 0.81]
Chapron et al. 2010	17	24	4.8%	0.71 [0.49; 0.87]
Hung et al. 2020	3	5	1.1%	0.60 [0.15; 0.95]
Langmade 1975	2	5	1.1%	0.40 [0.05; 0.85]
Malzoni et al. 2016	56	88	16.5%	0.64 [0.53; 0.74]
Nezhat et al. 1996	11	20	4.0%	0.55 [0.32; 0.77]
Seracchioli et al. 2015	49	70	13.3%	0.70 [0.58; 0.80]
Wang et al. 2015	54	79	14.9%	0.68 [0.57; 0.78]
Yamada et al. 2022	17	27	5.3%	0.63 [0.42; 0.81]
**Total (95% CI)**		**527**	**100.0%**	**0.70 [0.66; 0.75]**
Heterogeneity: $Tau^2 = 0.0002$; $Chi^2 = 12.34$, df = 12 (P = 0.4190); $I^2 = 2.7\%$				

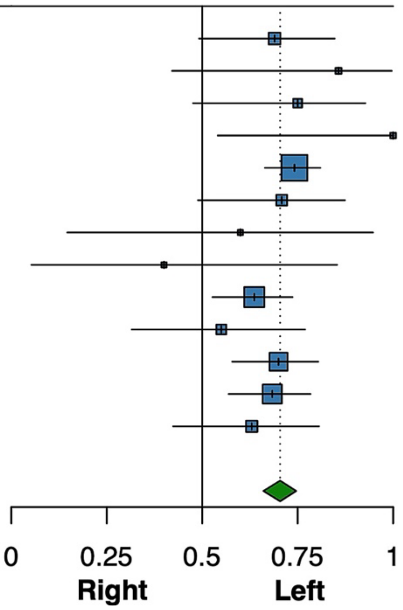

Supplementary Figure S3: Sensitivity analysis: meta-analysis of the proportions of left-sided ureteral lesions treated with excisional surgery.
Abbreviations: Random, random effect model; CI, Confidence Interval.

Study or Subgroup	Left Events	Total	Weight	IV, Random, 95% CI
**Timing = retrospective**				
Al–Khawaja et al. 2008	6	7	1.7%	0.86 [0.42; 1.00]
Antonelli et al. 2006	12	16	3.0%	0.75 [0.48; 0.93]
Azioni et al. 2010	6	6	1.5%	1.00 [0.54; 1.00]
Bosev et al. 2009	56	96	6.3%	0.58 [0.48; 0.68]
Chapron et al. 2010	17	24	3.7%	0.71 [0.49; 0.86]
Darwish et al. 2017	17	34	4.4%	0.50 [0.32; 0.68]
Frenna et al. 2007	18	30	4.2%	0.60 [0.41; 0.77]
Hung et al. 2020	3	5	1.3%	0.60 [0.18; 0.91]
Knabben et al. 2015	54	69	5.7%	0.78 [0.67; 0.88]
Kwok et al. 2020	22	40	4.7%	0.55 [0.38; 0.71]
Langebrekke and Qvigstad 2011	2	3	0.9%	0.67 [0.12; 0.97]
Langmade 1975	2	5	1.3%	0.40 [0.11; 0.81]
Miranda–Mendoza et al. 2012	10	12	2.5%	0.83 [0.52; 0.96]
Nezhat et al. 1996	11	20	3.4%	0.55 [0.27; 0.83]
Pérez–Utrilla Pérez et al. 2009	2	5	1.3%	0.40 [0.05; 0.85]
Pugliese et al. 2006	2	3	0.9%	0.67 [0.17; 0.92]
Seracchioli et al. 2008	14	22	3.6%	0.64 [0.40; 0.84]
Seracchioli et al. 2015	49	70	5.8%	0.70 [0.58; 0.81]
Sillou et al. 2015	19	24	3.7%	0.79 [0.59; 0.97]
Uccella et al. 2014	36	43	4.9%	0.84 [0.74; 1.00]
Vercellini et al. 2000	6	6	1.5%	1.00 [0.54; 0.98]
Wang et al. 2015	54	79	6.0%	0.68 [0.57; 0.78]
Yamada et al. 2022	17	27	4.0%	0.63 [0.42; 0.81]
**Total (95% CI)**		**646**	**76.0%**	**0.69 [0.64; 0.75]**
Heterogeneity: $Tau^2 = 0.0066$; $Chi^2 = 39.02$, df = 22 (P = 0.0140); $I^2 = 43.6\%$				
**Timing = Prospective**				
Ceccaroni et al. 2019	112	151	6.8%	0.74 [0.67; 0.81]
Donnez et al. 2002	10	17	3.1%	0.59 [0.33; 0.81]
Ghezzi et al. 2006	24	29	4.1%	0.83 [0.66; 0.98]
Mereu et al. 2010	37	50	5.2%	0.74 [0.60; 0.85]
Soriano et al. 2011	37	41	4.8%	0.90 [0.77; 0.98]
**Total (95% CI)**		**288**	**24.0%**	**0.78 [0.69; 0.86]**
Heterogeneity: $Tau^2 = 0.0066$; $Chi^2 = 8.87$, df = 4 (P = 0.0645); $I^2 = 54.9\%$				
**Total (95% CI)**		**934**	**100.0%**	**0.72 [0.67; 0.77]**
Heterogeneity: $Tau^2 = 0.0087$; $Chi^2 = 55.95$, df = 27 (P = 0.0009); $I^2 = 51.7\%$				
Test for subgroup differences: $Chi^2 = 3.19$, df = 1 (P = 0.0740)				

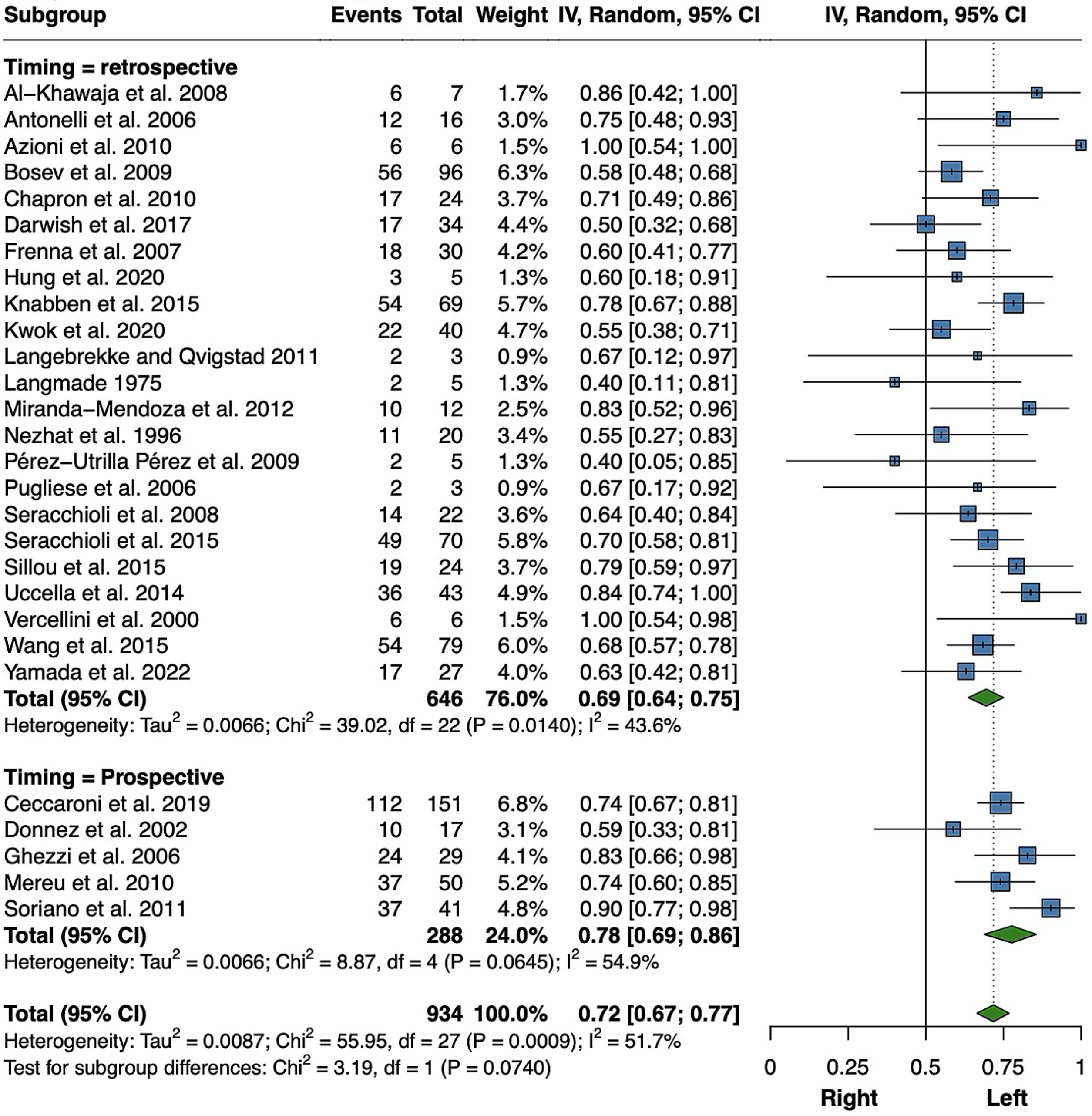

Supplementary Figure S4: Sensitivity analysis: meta-analysis of the proportions of left-sided ureteral lesions among explicitly specified unilateral lesions.

Abbreviations: Random, random effect model; CI, Confidence Interval.

Study	Left Events	Total	Weight	IV, Random, 95% CI
Anaf et al. 2009	20	23	5.8%	0.87 [0.66; 0.97]
Bailey et al. 1994	10	12	4.9%	0.83 [0.52; 0.98]
Chapron et al. 2006	30	48	6.6%	0.62 [0.47; 0.76]
Dobó et al. 2023	16	24	5.9%	0.67 [0.45; 0.84]
Dousset et al. 2010	23	36	6.3%	0.64 [0.46; 0.79]
Fleisch et al. 2005	4	5	3.4%	0.80 [0.28; 0.99]
Hernández Gutiérrez et al. 2019	5	14	5.1%	0.36 [0.13; 0.65]
Ianieri et al. 2024	24	26	6.0%	0.92 [0.75; 0.99]
Keckstein et al. 2005	32	52	6.6%	0.62 [0.47; 0.75]
Marcellin et al. 2019	73	118	7.1%	0.62 [0.52; 0.71]
Martinez–Serrano et al. 2015	25	39	6.4%	0.64 [0.47; 0.79]
Minelli et al. 2009	35	35	6.3%	1.00 [0.90; 1.00]
Mohr et al. 2005	12	19	5.6%	0.63 [0.38; 0.84]
Pereira et al. 2009	64	106	7.0%	0.60 [0.50; 0.70]
Prystowsky et al. 1988	4	17	5.4%	0.24 [0.07; 0.50]
Urbach et al. 1998	9	18	5.5%	0.50 [0.26; 0.74]
Yantiss et al. 2001	18	29	6.1%	0.62 [0.42; 0.79]
**Total (95% CI)**		**621**	**100.0%**	**0.68 [0.57; 0.78]**

Heterogeneity: $Tau^2 = 0.0381$; $Chi^2 = 81.98$, df = 16 ($P < 0.0001$); $I^2 = 80.5\%$

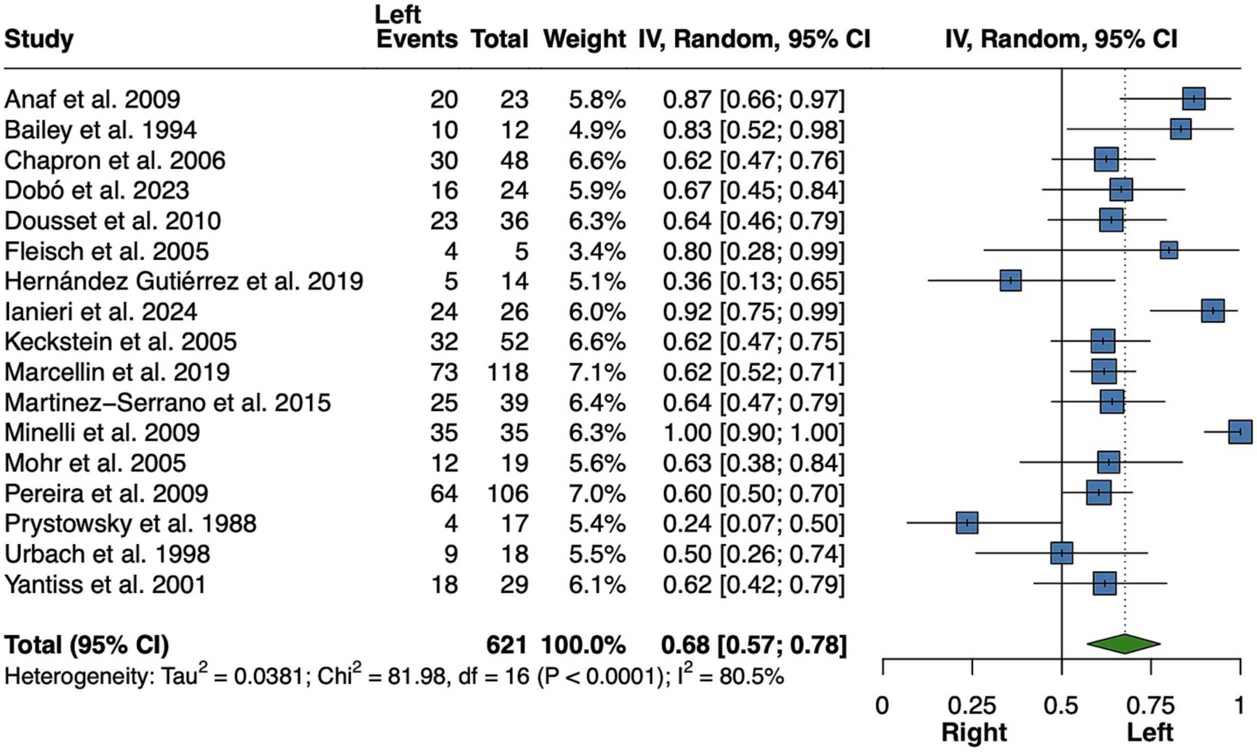

Supplementary Figure S5: Sensitivity analysis: meta-analysis of the proportions of left-sided bowel lesions treated with segmental resection.

Abbreviations: Random, random effect model; CI, Confidence Interval.

Study or Subgroup	Left Events	Total	Weight	IV, Random, 95% CI
**Timing = retrospective**				
Anaf et al. 2009	20	23	12.1%	0.87 [0.66; 0.97]
Chapron et al. 2006	30	48	15.7%	0.62 [0.47; 0.76]
Fleisch et al. 2005	4	5	4.9%	0.80 [0.31; 0.95]
Ianieri et al. 2024	24	26	12.8%	0.92 [0.75; 1.00]
Marcellin et al. 2019	73	118	18.8%	0.62 [0.53; 0.74]
Mohr et al. 2005	12	19	11.1%	0.63 [0.39; 0.83]
**Total (95% CI)**		**239**	**75.4%**	**0.74 [0.61; 0.86]**
Heterogeneity: $Tau^2 = 0.0187$; $Chi^2 = 16.4$, df = 5 ($P = 0.0058$); $I^2 = 69.5\%$				
**Timing = Prospective**				
Dousset et al. 2010	23	36	14.4%	0.64 [0.46; 0.79]
Faccioli et al. 2010	13	16	10.2%	0.81 [0.54; 0.96]
**Total (95% CI)**		**52**	**24.6%**	**0.71 [0.54; 0.85]**
Heterogeneity: $Tau^2 = 0.0049$; $Chi^2 = 1.45$, df = 1 ($P = 0.2288$); $I^2 = 31\%$				
**Total (95% CI)**		**291**	**100.0%**	**0.73 [0.63; 0.83]**

Heterogeneity: $Tau^2 = 0.0133$; $Chi^2 = 17.85$, df = 7 ($P = 0.0127$); $I^2 = 60.8\%$

Test for subgroup differences: $Chi^2 = 0.11$, df = 1 ($P = 0.7428$)

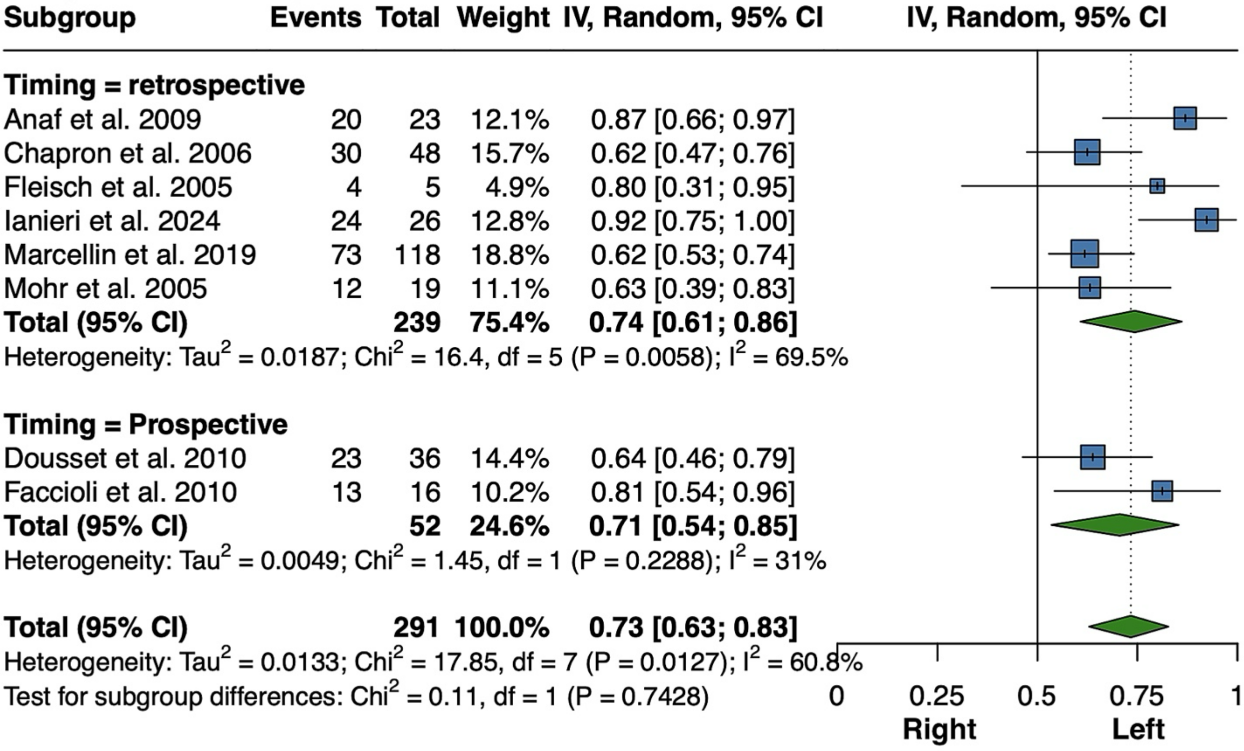

Supplementary Figure S6: Sensitivity analysis: meta-analysis of the proportions of left-sided bowel lesions among explicitly specified unilateral lesions.

Abbreviations: Random, random effect model; CI, Confidence Interval.

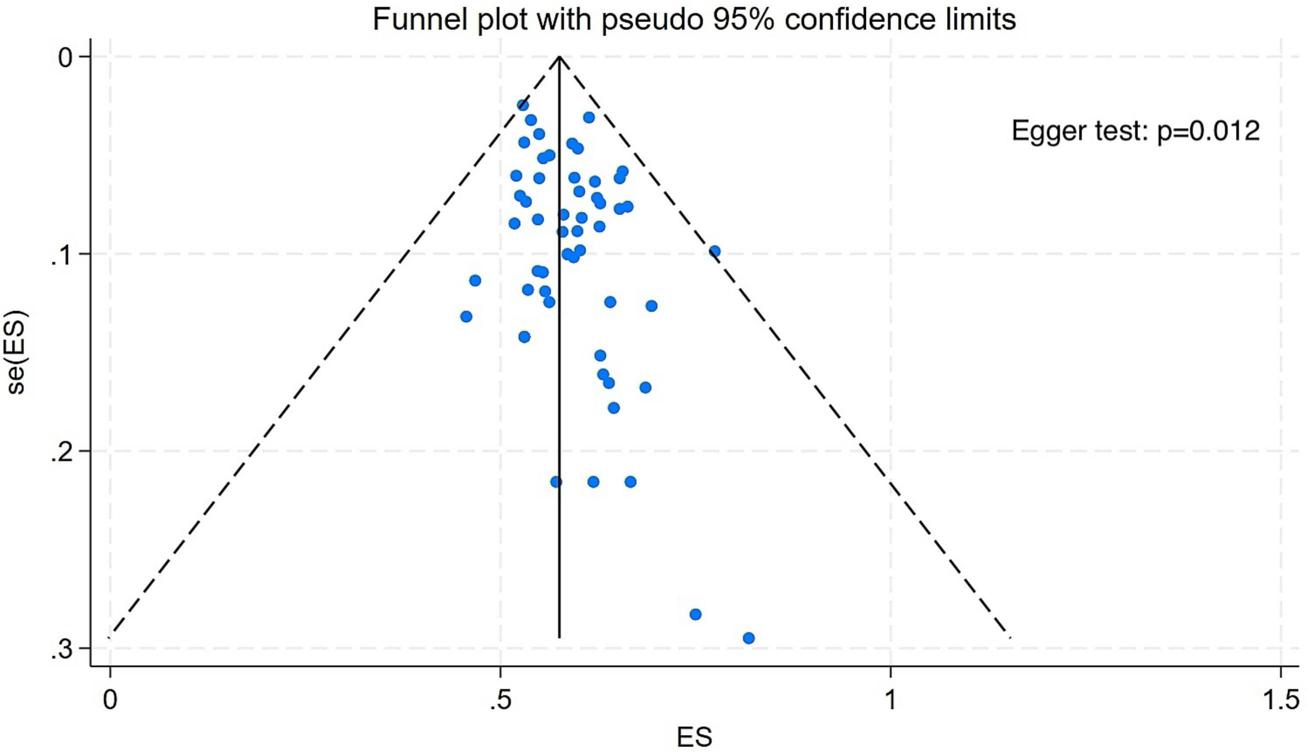

Supplementary Figure S7: Funnel plot for studies assessing the lateral distribution of ovarian endometriomas.

Abbreviations: se, standard error; ES, Effect Size.

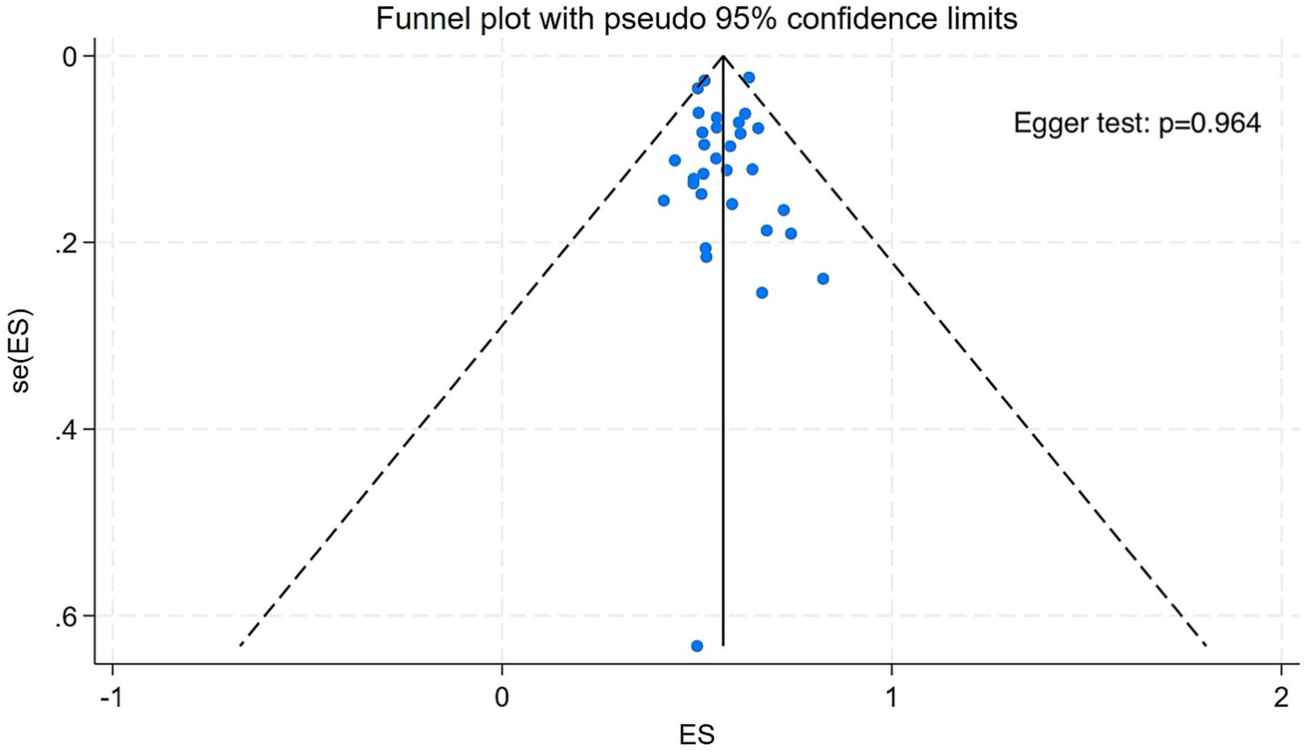

Supplementary Figure S8: Funnel plot for studies assessing the lateral distribution of endometriotic uterosacral ligament lesions.

Abbreviations: se, standard error; ES, Effect Size.

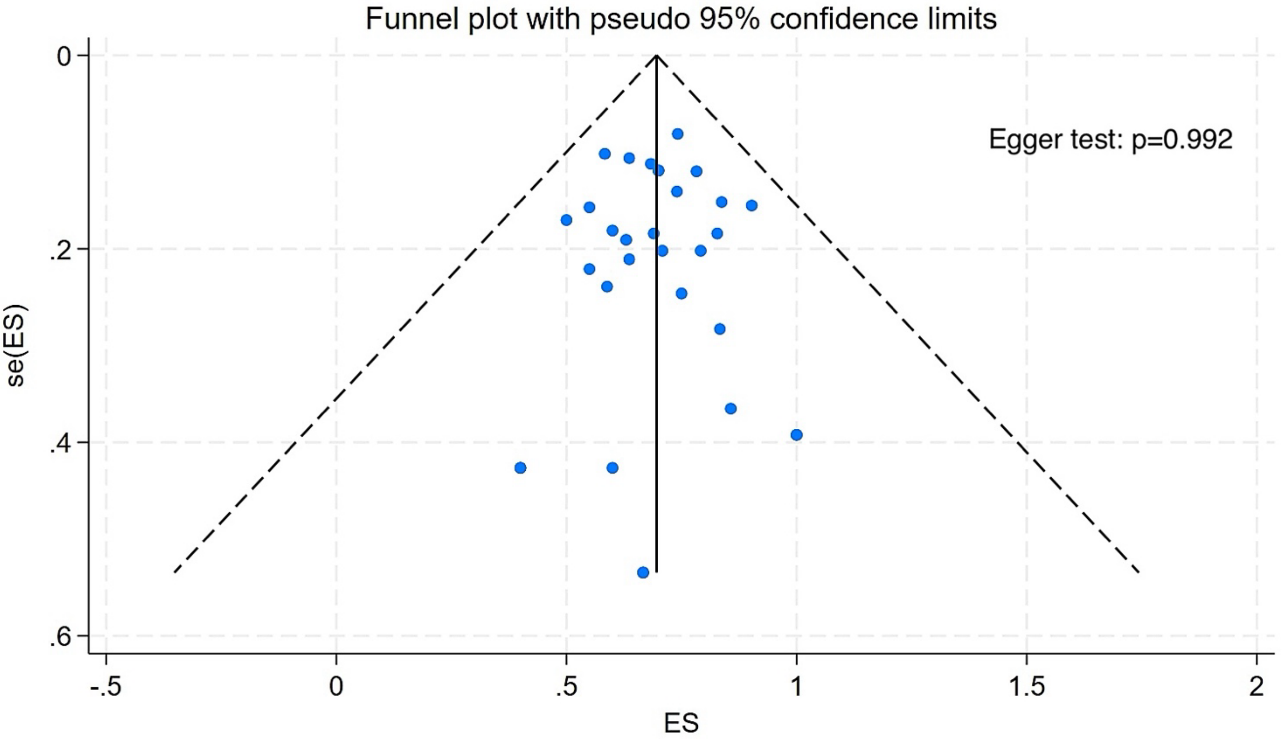

Supplementary Figure S9: Funnel plot for studies assessing the lateral distribution of endometriotic ureteral lesions.
Abbreviations: se, standard error; ES, Effect Size.

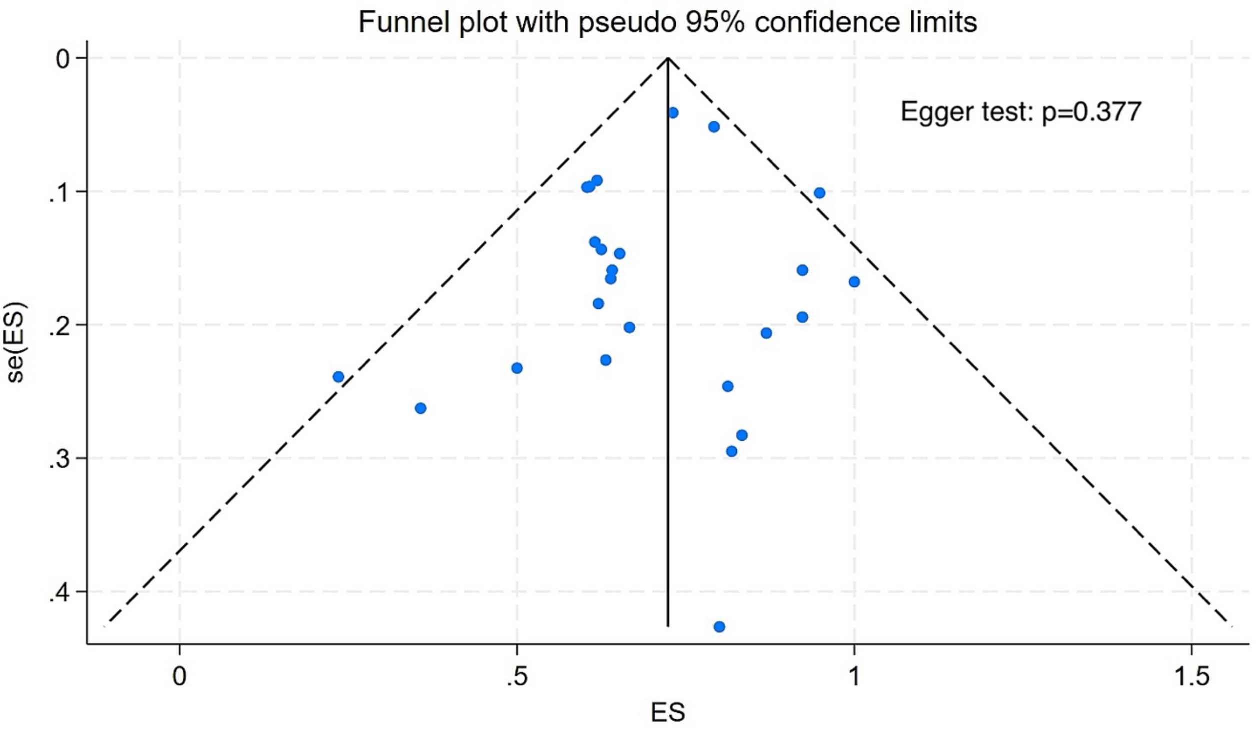

Supplementary Figure S10: Funnel plot for studies assessing the lateral distribution of endometriotic bowel lesions.
Abbreviations: se, standard error; ES, Effect Size.

Study or Subgroup	Right Events	Total	Weight	IV, Random, 95% CI
**Timing = retrospective**				
Attaran et al. 2013	5	5	2.0%	1.00 [0.48; 1.00]
Bobbio et al. 2024	18	20	6.4%	0.90 [0.68; 0.99]
Duyos et al. 2013	2	2	0.9%	1.00 [0.16; 1.00]
Ezemba et al. 2021	12	12	4.3%	1.00 [0.74; 1.00]
Flieder et al. 1998	4	4	1.7%	1.00 [0.40; 1.00]
Furuta et al. 2018	3	3	1.3%	1.00 [0.29; 1.00]
Ghigna et al. 2015	4	4	1.7%	1.00 [0.40; 1.00]
Haga et al. 2014	91	92	17.2%	0.99 [0.94; 1.00]
Legras et al. 2014	30	31	9.0%	0.97 [0.83; 1.00]
Marshall et al. 2005	2	2	0.9%	1.00 [0.16; 1.00]
Ochi et al. 2022	159	160	21.5%	0.99 [0.97; 1.00]
Redwine et al. 2002	7	7	2.7%	1.00 [0.58; 0.97]
Rousset–Jablonski et al. 2011	45	46	11.7%	0.98 [0.92; 1.00]
**Total (95% CI)**		**388**	**81.4%**	**1.00 [1.00; 1.00]**
Heterogeneity: $Tau^2 = 0.0010$; $Chi^2 = 7.13$, df = 12 (P = 0.8491); $I^2 = 0\%$				
**Timing = Prospective**				
Pagano et al. 2023	60	65	14.4%	0.92 [0.83; 0.98]
Tulandi et al. 2018	12	12	4.3%	1.00 [0.74; 1.00]
**Total (95% CI)**		**77**	**18.6%**	**0.95 [0.88; 0.99]**
Heterogeneity: $Tau^2 = 0$; $Chi^2 = 0.97$, df = 1 (P = 0.3245); $I^2 = 0\%$				
**Total (95% CI)**		**465**	**100.0%**	**1.00 [0.99; 1.00]**
Heterogeneity: $Tau^2 = 0.0030$; $Chi^2 = 11.94$, df = 14 (P = 0.6115); $I^2 = 0.0\%$				
Test for subgroup differences: $Chi^2 = 3.08$, df = 1 (P = 0.0795)				

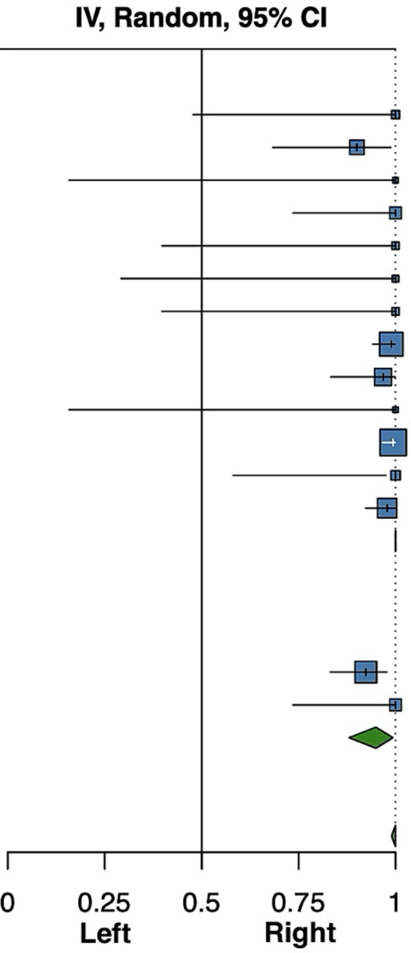

Supplementary Figure S11: Sensitivity analysis: meta-analysis of the proportion of right-sided thoracic lesions including patients with pleural endometriotic lesions only or studies explicitly stating that the entire bilateral hemidiaphragms were examined.

Abbreviations: Random, random effect model; CI, Confidence Interval.

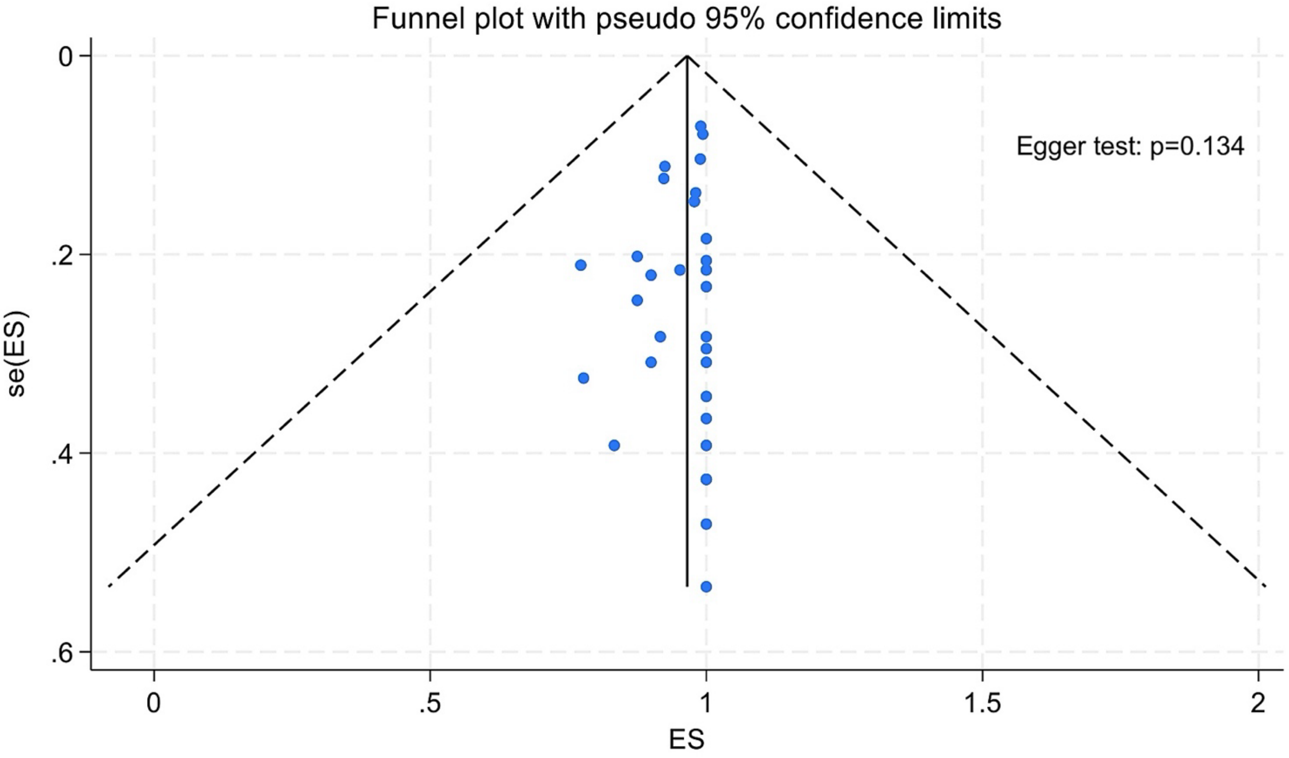

Supplementary Figure S12: Funnel plot for studies assessing the lateral distribution of endometriotic thoracic lesions.
Abbreviations: se, standard error; ES, Effect Size.

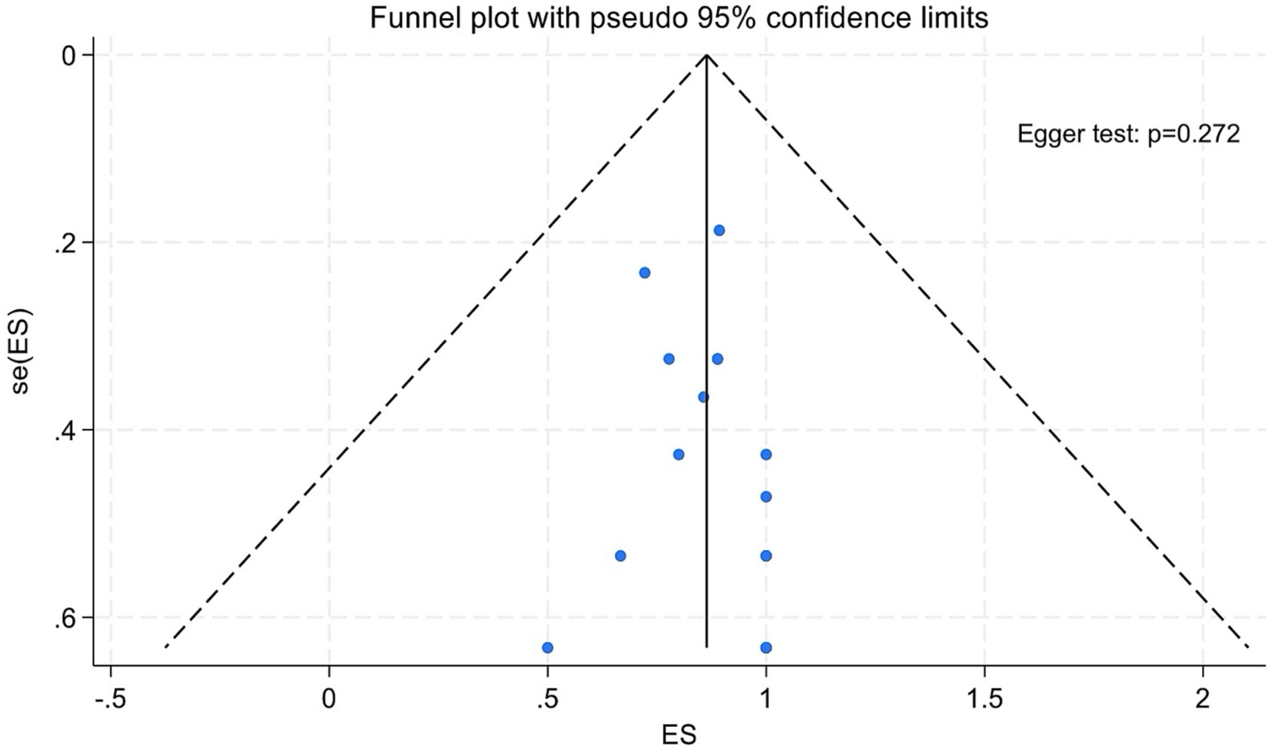

Supplementary Figure S13: Funnel plot for studies assessing the lateral distribution of endometriotic inguinal lesions.
Abbreviations: se, standard error; ES, Effect Size.
