# Supplementary material for: Lateral distribution of endometriotic lesions: the anatomical recesses hypothesis. A systematic review and meta-analysis
Source: Hum Reprod Open. 2025 Oct 24;2026(1):hoaf064. doi: 10.1093/hropen/hoaf064 (PMC12816922; doi:10.1093/hropen/hoaf064)
Supplement: hoaf064_Supplementary_Data [file hoaf064_supplementary_data.zip › Supplementary File S1. Search strategy.docx]

**Search strategy**

**PubMed search**

((endometriosis[MeSH Terms]) AND ((lateral*) OR (bilateral) OR (right) OR (left) OR (umbilic*) OR (asymmetr*) OR (distribution))) OR ((endometriosis) AND ((intestinal surgery) OR (bowel surgery))) AND (english[Filter])

**Embase search**

(('endometriosis'/exp OR endometriosis) AND (lateral* OR bilateral OR right OR left OR umbilic* OR asymmetr* OR 'distribution'/exp OR distribution) OR (('endometriosis'/exp OR endometriosis) AND ('intestinal surgery'/exp OR 'intestinal surgery' OR (intestinal AND ('surgery'/exp OR surgery)) OR 'bowel surgery' OR (('bowel'/exp OR bowel) AND ('surgery'/exp OR surgery))))) AND [english]/lim
