# Supplementary material for: Lateral distribution of endometriotic lesions: the anatomical recesses hypothesis. A systematic review and meta-analysis
Source: Hum Reprod Open. 2025 Oct 24;2026(1):hoaf064. doi: 10.1093/hropen/hoaf064 (PMC12816922; doi:10.1093/hropen/hoaf064)
Supplement: hoaf064_Supplementary_Data [file hoaf064_supplementary_data.zip › Supplementary Table S1.docx]

**Supplementary Table S1.** Main characteristics of the selected studies evaluating the lateral distribution of ovarian endometriomas.

| **Author,** **year** | **Country** | **Study design** | **Age**  **(mean ± SD)** | **No of patients with left lesion** | **No of patients with right lesion** | **Sum of patients with unilateral lesion** | **No of patients with bilateral lesion** |
| --- | --- | --- | --- | --- | --- | --- | --- |
| Abbott *et al.*  (2003) | Australia | Prospective (cohort) | 31 (20-48)^a^ | 24 | 11 | 35 | 16 |
| Abo *et al.*  (2018) | France | Retrospective (from cohort) | 33 ± 6.5 | 142 | 131 | 273^b^ | |
| Al-Fozan and Tulandi  (2003) | Canada | Retrospective (case series) | 33.5 ± 0.3 | 90 | 59 | 149 | 36 |
| Alborzi *et al.*  (2017) | Iran | Retrospective (from cohort) | 31.2 ± 6.3 | 355 | 291 | 646 | 322 |
| Araujo *et al.*  (2021) | US | Retrospective (case series) | 31 ± 6.3 | 46 | 38 | 84 | 0 |
| Ari *et al.*  (2023) | Turkey | Retrospective (from cohort) | 31 (21-46)^a^ | 73 | 53 | 126 | 46 |
| Audebert *et al.*  (2018) | Greece | Retrospective (from cohort) | 33 (15-63)^a^ | 224 | 174 | 398 | 87 |
| Bazi *et al.*  (2007) | Lebanon | Retrospective (case series) | 34.9 ± 8.9 | 57 | 39 | 96 | 34 |
| Bhurke *et al.*  (2022) | US | Prospective (cohort) | 28.9 ± 3.7 | 102 | 91 | 193^b^ | |
| Bindra *et al.*  (2023) | India | Retrospective (from cohort) | 32 (20-42)^a^ | 29 | 21 | 50 | 14 |
| Bosev *et al.*  (2009) | Canada | Retrospective (case series) | 34 (19-52)^a^ | 26 | 23 | 49 | 47 |
| Bouaziz *et al.*  (2017) | Israel | Retrospective (case series) | 31 ± 4.5 | 32 | 23 | 55 | 14 |
| Ceccaroni *et al.*  (2019) | Italy | Prospective  (cohort) | 36 (18-50)^a^ | 58 | 41 | 99^b^ | |
| Chapron *et al.*  (2001) | France | Retrospective (case series) | left 30.4±5.7; right 32.5±6.4 | 9 | 2 | 11 | 0 |
| Chopin *et al.*  (2006) | France | Retrospective (from cohort) | Gr1 35.1±7.8; Gr2 34.9±7.4 | 114 | 58 | 172 | 67 |
| Ciavattini *et al.*  (2004) | Italy | Retrospective (case series) | 34.4 ± 7.8 | 88 | 53 | 141 | 0 |
| Coccia *et al.*  (2011) | Italy | Prospective (cohort) | 32.6 ± 5.6 | 90 | 65 | 155 | 84 |
| Dafna *et al.*  (2019) | Israel | Retrospective (from cohort) | Gr emergent 33.9±11.1;  Gr elective 39±10.9 | 105 | 95 | 200 | 25 |
| Di Giovanni *et al.*  (2023) | Austria | Retrospective (from cohort) | 37.3 ± 6.6 | 39 | 31 | 70^b^ | |
| Ercan *et al.*  (2011) | Turkey | Prospective (cohort) | 29.4 ± 4.6 | 23 | 13 | 36 | 64 |
| Ferrero *et al.*  (2005) | Italy | Retrospective (case series) | 34.8 ± 7.5 | 275 | 184 | 459 | 110 |
| Ghezzi *et al.*  (2001) | Italy | Retrospective (cross-sectional) | left 29.5±4.5; right 31.4±6.4; bilateral 32.8±6.9 | 58 | 41 | 99 | 22 |
| Harada *et al.*  (2015) | Japan | Retrospective (from cohort) | 37 ± 3.4 | 14 | 7 | 21 | 0 |
| Hudelist *et al.*  (2009) | Austria | Prospective (cohort) | 33 (16-45)^a^ | 26 | 23 | 49 | 0 |
| Jenkins *et al.*  (1986) | US | Retrospective (case series) | NR | 43 | 19 | 62 | 38^c^ |
| Khan *et al.*  (2013) | Japan | Retrospective (from cohort) | 20-39 | 154 | 94 | 248 | 102 |
| Kikuchi *et al.*  (2006) | Japan | Retrospective (from cohort) | 31 ± 5 | 128 | 85 | 213 | 102 |
| Kwok *et al.*  (2020) | China | Retrospective (from cohort) | 17-49 | 98 | 86 | 184 | 120 |
| Lee *et al.*  (2013) | Korea | Retrospective (from cohort) | left 34.7±6.5; right 31.5±6.2; bilateral 33.8±6 | 866 | 772 | 1638 | 576 |
| Li *et al.*  (2024) | China | Retrospective (case series) | 27.5 ± 3.8 | 27 | 16 | 43 | 3 |
| Liu *et al.*  (2008) | China | Retrospective (from cohort) | 21.4 ± 7.3 | 208 | 167 | 375 | 217 |
| Matalliotaki *et al.*  (2020) | Greece | Retrospective (from cohort) | Gr1 32.5±6.6; Gr2 36.3±8.2 | 193 | 101 | 294 | 65 |
| Mereu *et al.*  (2010) | Italy | Prospective (cohort) | 32.7 ± 4 | 12 | 9 | 21 | 3 |
| Mereu *et al.*  (2012) | Italy | Prospective (cohort) | 33.2 ± 6.5 | 48 | 23 | 71 | 28 |
| Meuleman *et al.*  (2009) | Belgium | Retrospective (from cohort) | 32 (24-42)^a^ | 9 | 3 | 12 | 10 |
| Moro *et al.*  (2024) | Italy | Prospective (cohort) | 35 (31-42)^a^ | 72 | 67 | 139^b^ | |
| Nicolaus *et al.*  (2020) | Germany | Retrospective (from cohort) | 34 ± 4.4 | 26 | 31 | 57^b^ | |
| Özyer *et al.*  (2013) | Turkey | Retrospective (case series) | 22 ± 2 | 36^d^ | 41^d^ | 77^d^ | 14^d^ |
| Pagano *et al.*  (2023) | Switzerland | Prospective (cohort) | 34 (19-40)^a^ | 41 | 23 | 64 | 10 |
| Parazzini  (2003) | Italy | Retrospective (cross-sectional) | 34 (16-54)^a^ | 94 | 79 | 173 | 0^e^ |
| Porpora *et al.*  (2014) | Italy | Prospective (case-control) | (20-45)^a^ | 13 | 8 | 21 | 21 |
| Prefumo *et al.*  (2002) | Italy | Retrospective (case series) | 35.8 (14-78)^a^ | 171 | 91 | 262 | 63 |
| Redwine  (1999) | US | Retrospective (from cohort) | NR | 303 | 209 | 512^b^ | |
| Roman *et al.*  (2010) | France | Retrospective (from cohort) | 29.3 ± 5.3 | 24 | 14 | 38 | 8 |
| Roman *et al.*  (2020) ^f^ | France | Retrospective (from cohort) | NR | 518^g^ | 443^g^ | 961^b,g^ | |
| Serracchioli *et al.*  (2014) | Italy | Prospective  (RCT) | Gr1 33.2±4.3; G2 33.9±4.4 | 38 | 33 | 71 | 9 |
| Sesti *et al.*  (2009) | Italy | Prospective  (RCT) | Gr 1 31.3±5.1; Gr2 30.8±6; Gr3 30.3±5.6; Gr4 29.3±5.7 | 121 | 73 | 194 | 46 |
| Signorile *et al.*  (2022) | Italy | Retrospective (from cohort) | 20-60 | 279 | 247 | 526^b^ | |
| Song *et al.*  (2016) | China | Retrospective (case series) | NR | 20 | 11 | 31 | 10 |
| Sznurkowki and Emerich  (2008) | Poland | Retrospective (case series) | 37.8 ± 8.4 | 113 | 67 | 180 | 54 |
| Ulukus *et al.*  (2012) | Turkey | Retrospective (case series) | 29.5 ± 5.8 | 109 | 58 | 167 | 72 |
| Vercellini *et al.*  (1998) | Italy | Retrospective (case series) | 31 ± 5 | 641^h^ | 404^h^ | 1045^h^ | 362^h^ |
| Yu *et al.*  (2015) | Taiwan | Retrospective (from cohort) | 34.3 ± 4 | 62 | 41 | 103^b^ | |
| Yuan *et al.*  (2014) | China | Retrospective (from cohort) | 32.4 ± 7.2 | 157 | 107 | 264 | 111 |

^a^ Range (min-max) or (min-max) only.

^b^ Articles in which it is not expressly stated whether the total lesions considered are unilateral or bilateral.

^c^ All visualized intraoperatively but not specified surgery type.

^d^ Adolescents aged < 24 years.

^e^ This data was considered zero because 173 were definitely unilateral lesions, whereas 594 were bilateral lesions but included both ovarian cysts and pelvic peritoneal localisations.

^f^ This article may include some patients previously reported by Abo *et al.* (2018), but with a slightly different study period (Jun 2009-Dec 2015 vs Oct 2009-May 2019). Moreover, an earlier study by the same group (Roman *et al.,* 2010) was also included in this meta-analysis. Although some degree of overlap with their later publication (Roman *et al.,* 2020) cannot be excluded, the recruitment period for the 2010 study was not reported, and its inclusion was deemed appropriate.

^g^ Only 680 patients underwent surgery on the ovary, the remaining reported lesions are intraoperative findings.

^h^ Overall patient numbers, later published in Vercellini *et al.* (2002).

SD: Standard Deviation

NR: Not Reported

RCT: Randomized Clinical Trial
