# Supplementary material for: Lateral distribution of endometriotic lesions: the anatomical recesses hypothesis. A systematic review and meta-analysis
Source: Hum Reprod Open. 2025 Oct 24;2026(1):hoaf064. doi: 10.1093/hropen/hoaf064 (PMC12816922; doi:10.1093/hropen/hoaf064)
Supplement: hoaf064_Supplementary_Data [file hoaf064_supplementary_data.zip › Supplementary Table S2.docx]

**Supplementary Table S2.** Main characteristics of the selected studies evaluating the lateral distribution of endometriotic uterosacral ligament lesions.

| **Author,** **year** | **Country** | **Study design** | **Age**  **(mean ± SD)** | **No of patients with left lesion** | **No of patients with right lesion** | **Sum of patients with unilateral lesion** | **Sum of patients with bilateral lesion** | **Type of surgery** |
| --- | --- | --- | --- | --- | --- | --- | --- | --- |
| Abbott *et al.*  (2003) | Australia | Prospective (cohort) | 31 (20-48)^a^ | 17 | 24 | 41 | 76 | Not specified; histologically confirmed |
| Abdalla Ribeiro *et al.*  (2021) | Brazil | Retrospective (from cohort) | 35.5 ± 6 | 11 | 10 | 21 | 18 | Complete USL excision |
| Abo *et al.*  (2018) | France | Retrospective (from cohort) | 33 ± 6.5 | 62 | 44 | 106^b^ | | Nodule excision |
| Alborzi *et al.*  (2017) | Iran | Retrospective (from cohort) | 31.2 ± 6.3 | 414 | 411 | 825^b^ | | Complete USL excision |
| Araujo *et al.*  (2021) | US | Retrospective (case series) | 31 ± 6.3 | 45 | 37 | 82 | 10 | Complete USL excision |
| Ari *et al.*  (2023) | Turkey | Retrospective (from cohort) | 31 (21-46)^a^ | 35 | 44 | 79^b^ | | Complete USL excision |
| Audebert *et al.*  (2018) | Greece | Retrospective (from cohort) | 33 (15-60)^a^ | 119 | 77 | 196 | 272 | Not specified; histologically confirmed |
| Bazot *et al.*  (2012) | France | Retrospective (from cohort) | 34.1± 7.4 | 12 | 11 | 23^b^ | | Not specified; histologically confirmed |
| Bhurke *et al.*  (2022) | US | Prospective (cohort) | 28.9 ± 3.7 | 125 | 102 | 227^b^ | | Not specified; histologically confirmed |
| Bouaziz *et al.*  (2017) | Israel | Retrospective (case series) | 31 ± 4.5 | 28 | 18 | 57 | 11 | Not specified; all visualised intraoperatively |
| Ceccaroni *et al.*  (2019) | Italy | Prospective  (cohort) | 36 (18-50)^a^ | 93 | 76 | 169^b^ | | Complete USL excision |
| Chapron *et al.*  (2006) | France | Retrospective (from cohort) | 31.8 ± 5.6 | 109 | 57 | 166 | 56 | Not specified; histologically confirmed |
| Ciavattini *et al.*  (2004) | Italy | Retrospective (case series) | 34.4 ± 7.8 | 10 | 5 | 15 | 0 | Not specified; histologically confirmed |
| Di Giovanni *et al.*  (2023) | Austria | Retrospective (from cohort) | 37.3 ± 6.6 | 23 | 22 | 45 | 48 | Complete USL excision |
| Freger *et al.*  (2024) | Australia | Prospective (cohort) | 35.2 ± 7.2 | 14 | 3 | 17 | 9 | All lesions resected or biopsied |
| Hudelist *et al.*  (2009) | Austria | Prospective (cohort) | 33 (16-45)^a^ | 43 | 24 | 67 | 0 | Complete USL excision |
| Jenkins *et al.*  (1986) | US | Retrospective (case series) | NR | 38 | 28 | 66^b^ | | Not specified; all visualised intraoperatively |
| Kovoor *et al.*  (2011) | France | Retrospective (case series) | 36.5 ± 4.8 | 1 | 1 | 2 | 1 | Complete USL excision |
| Kwok *et al.*  (2020) | China | Retrospective (from cohort) | 17-49 | 57 | 53 | 110 | 135 | Not specified; histologically confirmed |
| Malzoni *et al.*  (2016) | Italy | Prospective (cohort) | 35.6 ± 4.7 | 88 | 56 | 144 | 104 | Not specified; all visualised intraoperatively |
| Mereu *et al.*  (2010) | Italy | Prospective (cohort) | 32.7 ± 4 | 20 | 7 | 27 | 24 | Complete USL excision |
| Mereu *et al.*  (2012) | Italy | Prospective (cohort) | 33.2 ± 6.5 | 32 | 30 | 62^b^ | | Complete USL excision |
| Moro *et al.*  (2024) | Italy | Prospective (cohort) | 35 (31-42) | 135 | 133 | 268^b^ | | Nodules or fibrosis treated surgically; USL excision in severe cases |
| Nicolaus *et al.*  (2020) | Germany | Retrospective (from cohort) | 34 ± 4.4 | 76 | 72 | 148^b^ | | Resection, coagulation or adhesiolysis; histologically confirmed |
| Qju *et al.*  (2023) | China | Prospective (cohort) | 36 ± 7 | 23 | 16 | 39^b^ | | Not specified; histologically confirmed |
| Redwine  (1999) | US | Retrospective (from cohort) | NR | 744 | 689 | 1433 | | Surgically treated, but type of surgery not specified |
| Roman *et al.*  (2020) ^c^ | France | Retrospective (from cohort) | NR | 162 | 98 | 260 | 40 | Not specified; histologically confirmed |
| Signorile *et al.*  (2022) | Italy | Retrospective (from cohort) | (20-60)^a^ | 1169 | 678 | 1847^b^ | | Clinical evaluation of which 1/4 confirmed by surgery and histology |
| Stoppa *et al.*  (2023) | France | Retrospective (cross-sectional) | 30.5 ± 7 | 26 | 27 | 53 | 14 | Complete USL excision, nodule excision or biopsy; histologically confirmed |
| Zannoni *et al.*  (2017) | Italy | Prospective (cross-sectional) | 37 ± 5.3 | 19 | 9 | 28^b^ | | Nodule excision |

^a^ Range (min-max) or (min-max) only.

^b^ Articles in which it is not expressly stated whether the total lesions considered are unilateral or bilateral.

^c^ This article may include some patients previously reported by Abo *et al.* 2018, but with a slightly different study period (Jun 2009-Dec 2015 vs Oct 2009-May 2019).

SD: Standard Deviation

USL: Utero Sacral Ligaments

NR: Not Reported
