# Supplementary material for: Lateral distribution of endometriotic lesions: the anatomical recesses hypothesis. A systematic review and meta-analysis
Source: Hum Reprod Open. 2025 Oct 24;2026(1):hoaf064. doi: 10.1093/hropen/hoaf064 (PMC12816922; doi:10.1093/hropen/hoaf064)
Supplement: hoaf064_Supplementary_Data [file hoaf064_supplementary_data.zip › Supplementary Table S3.docx]

**Supplementary Table S3.** Main characteristics of the selected studies evaluating the lateral distribution of endometriotic ureteral lesions.

| **Author, year** | **Country** | **Study design** | **Age**  **(mean ± SD)** | **No of patients with left lesion** | **No of patients with right lesion** | **Sum of patients with unilateral lesion** | **Sum of patients with bilateral lesion** | **Type of surgery** |
| --- | --- | --- | --- | --- | --- | --- | --- | --- |
| Abdalla Ribeiro *et al.*  (2021) | Brazil | Retrospective (from cohort) | 35.5 ± 6 | 20 | 9 | 29^b^ | | All patients with concomitant excision of bladder endometriosis nodules; type of ureter surgery not specified |
| Al-Khawaja *et al.*  (2008) | US | Retrospective (case series) | 50.8 ± 12 | 6 | 1 | 7 | 0 | 2 nephrectomy, 3 distal ureterectomy with reimplantation, 1 ureteroureterostomy, 1 relief of ureteral obstruction by resection of pelvic endometrioma |
| Antonelli *et al.*  (2006) | Italy | Retrospective (from cohort) | 33.1 (22-48)^a^ | 12 | 4 | 16 | 3 | 6 ureterolysis, 2 ureteroureterostomy, 9 ureterectomy and ureterocystoneostomy (2 nephrectomies and 14 partial cystectomy) |
| Azioni *et al.*  (2010) | Italy | Retrospective (case series) | 33.6 ± 3.2 | 6 | 0 | 6 | 0 | Ureteroneocystostomy and double-J stent |
| Bosev *et al.*  (2009) | Canada | Retrospective (case series) | 34 (19-52)^a^ | 56 | 40 | 96 | 10 | 2 resection and ureteroneocystostomy, remaining ureterolysis with excision or ablation of lesions |
| Ceccaroni *et al.*  (2019) | Italy | Prospective (cohort) | 36.1 | 112 | 39 | 151 | 9 | Ureteroneocystostomy |
| Chapron *et al.*  (2010) | France | Retrospective (from cohort) | 32.7 ± 5.4 | 17 | 7 | 24 | 5 | 4 severe ureteral ureterolysis, remaining radical surgery |
| Darwish *et al.*  (2017) | France | Retrospective (from cohort) | 34.8 ± 6.7 | 17 | 17 | 34 | 8 | Advanced ureterolysis (78%) and ureteral resection followed by end-to-end anastomosis or ureteroneocystostomy (22%) |
| Donnez *et al.*  (2002) | Belgium | Prospective (cohort) | 28.1 (21-39)^a^ | 10 | 7 | 17 | 1 | 16 ureterolysis, 2 ureteral resection and uretero-ureterostomy |
| Frenna *et al.*  (2007) | France | Retrospective (case series) | 31 ± 4.7 | 18 | 12 | 30 | 8 | Ureterolysis |
| Ghezzi *et al.*  (2006) | Italy | Prospective (cohort) | 31 (20-43)^a^ | 24 | 5 | 29 | 4 | Ureterolysis; 1 partial wall resection of the ureter, 1 segmental ureteral resection with vescicopsoas hitch |
| Hung *et al.*  (2020) | China | Retrospective (case series) | 36.2 ± 6.4 | 3 | 2 | 5 | 0 | Segmental resection reconstructed through ureteroureterostomy or ureteroneocystostomy |
| Knabben *et al.*  (2015) | Switzerland | Retrospective (case series) | 33.3 (20-57)^a^ | 54 | 15 | 69 | 37 | Ureterolysis and 2 ureterocystoneostomy |
| Kwok *et al.*  (2020) | China | Retrospective (from cohort) | (17-49)^a^ | 22 | 18 | 40 | 7 | Not specified type of ureter surgery, but histologically proven |
| Langebrekke and Qvigstad  (2011) | Norway | Retrospective (case series) | 31.6 ± 1.6 | 2 | 1 | 3 | 0 | 1 nephrostomy with later double-J ureteral stent, 1 nephrectomy, 1 ureterolysis |
| Langmade  (1975) | US | Retrospective (case series) | (39-49)^a^ | 2 | 3 | 5 | 0 | Ureteral reimplantation |
| Malzoni *et al.*  (2016) | Italy | Prospective (cohort) | 35.6 ± 4.7 | 56 | 32 | 88^b^ | | 24 mono/bilateral ureteral stents, 8 ureteral reanastomosis and stents, 2 ureteral stents and bladder nodule excision, 54 with bladder nodule excision |
| Matalliotakis *et al.*  (2017) | Greece | Retrospective (case series) | 35.4 ± 6.3 | 49 | 21 | 70^b^ | | Not specified type of ureteral surgery, but histologically proven |
| Mereu *et al.*  (2010) | Italy | Prospective (cohort) | 32.7 ± 4 | 37 | 13 | 50 | 6 | 35 ureterolysis, 19 ureteroureterostomy, 2 nephrectomies |
| Miranda-Mendoza *et al.*  (2012) | Chile | Retrospective (case series) | 33 (24-48)^a^ | 10 | 2 | 12 | 1 | 7 ureterolysis, 6 segmental resections with end-to-end anastomosis |
| Nezhat *et al.*  (1996) | US | Retrospective (case series) | 35 (24-46)^a^ | 11 | 9 | 20 | 1 | 10 ureterolysis and excision of endometriosis, 7 partial wall resections (partial obstruction); 3 partial resection and ureteroureterostomy, 1 ureteroneocystostomy (complete obstruction) |
| Pérez-Utrilla Pérez *et al.*  (2009) | Spain | Retrospective (case series) | 37.7 (30-45)^a^ | 2 | 3 | 5 | 2 | 5 ureteroneocystostomy, 2 ureterolysis, 1 ureterolysis plus later ureteral resection and end-to-end anastomosis |
| Pugliese *et al.*  (2006) | US | Retrospective (case series) | 48 ±2.9 | 2 | 1 | 3 | 0 | 1 nephroureterectomy, 1 ureterolysis, 1 nephroureterectomy with partial cystectomy |
| Seracchioli *et al.*  (2008) | Italy | Retrospective (from cohort) | 33.3 ± 6.4 | 14 | 8 | 22 | 8 | 22 ureterolysis, 5 segmental ureteral resection and ureteroureterostomy, 3 ureterectomy and ureterocystoneostomy |
| Seracchioli *et al.*  (2015) | Italy | Retrospective (from cohort) | 35.1 ± 6.1 | 49 | 21 | 70 | 7 | 70 nodule ureteral excision, 14 ureteral resections |
| Sillou *et al.*  (2015) | France | Retrospective (cross-sectional) | 33.5 (26-45)^a^ | 19 | 5 | 24 | 7 | 18 ureterolysis, 15 ureteric or bladder resection-reimplantation, 8 nephro-ureterectomy |
| Soriano *et al.*  (2011) | Israel | Prospective (cohort) | 32.8 ± 5.4 | 37 | 4 | 41 | 4 | Ureterolysis, 4 primary reimplantation of the ureter |
| Uccella *et al.*  (2014) | Italy | Retrospective (from cohort) | 35 (20-54)a | 36^c^ | 7^c^ | 43^c^ | 23^c^ | Ureterolysis, 4 double-J stent, 1 uretero-ureteral reanastomosis;  hydronephrosis ^b^ |
| Vercellini *et al.*  (2000b) | Italy | Retrospective (case series) | (24-40)^a^ | 6 | 0 | 6 | 0 | 2 ureteroneocystostomy, 2 ureterolysis with dissection of the ureter, 1 ureteroureterostomy, 1 nephroureterectomy |
| Wang *et al.*  (2015) | China | Retrospective (from cohort) | (21-50)^a^ | 54 | 25 | 79 | 2 | Ureterectomy with ureteroureterostomy or ureterocystoneostomy |
| Yamada *et al.*  (2022) | Japan | Retrospective (from cohort) | 38.5 (25-51)^a^ | 17 | 10 | 27 | 2 | Ureteroneocystostomy |

^a^ Range (min-max) or (min-max) only.

^b^ Articles in which it is not expressly stated whether the total lesions considered are unilateral or bilateral.

^c^ Diagnosis of right or left laterality performed preoperatively with assessment of hydronephrosis; all lesions were subsequently confirmed and treated surgically.

SD: Standard Deviation
