# Supplementary material for: Lateral distribution of endometriotic lesions: the anatomical recesses hypothesis. A systematic review and meta-analysis
Source: Hum Reprod Open. 2025 Oct 24;2026(1):hoaf064. doi: 10.1093/hropen/hoaf064 (PMC12816922; doi:10.1093/hropen/hoaf064)
Supplement: hoaf064_Supplementary_Data [file hoaf064_supplementary_data.zip › Supplementary Table S4.docx]

**Supplementary Table S4.** Main characteristics of the selected studies evaluating the lateral distribution of endometriotic bowel lesions.

| **Author, year** | **Country** | **Study design** | **Age**  **(mean ± SD)** | **No of patients with left lesion and intestinal site** | **No of patients with right lesion and intestinal site** | **Sum of patients with unilateral lesion** | **No of patients with bilateral lesion** | **Type of surgery** |
| --- | --- | --- | --- | --- | --- | --- | --- | --- |
| Abo *et al.*  (2018) | France | Retrospective (from cohort) | 33 ± 6.5 | 30  sigma | 16  ileocecal junction | 46^b^ | | 42 bowel resections, 4 disc excision |
| Anaf *et al.*  (2009) | Belgium | Retrospective (from cohort) | 33 ± 0.5 | 20  sigma | 3  ileocecal junction | 23 | 3 | Resection |
| Audebert *et al.*  (2018) | Greece | Retrospective (from cohort) | 33 (15-63)^a^ | 92  sigma | 5  caecum / appendix | 97^b^ | | Superficial intestinal implants, not specified type of surgery but all histologically confirmed |
| Bailey *et al.*  (1994) | US | Retrospective (case series) | 33 (22-48)^a^ | 10  sigma | 2  caecum | 12^b^ | | Resection |
| Buffeteau *et al.*  (2023) | France | Retrospective (from cohort) | 36 ± 5.1 | 36  sigma | 3  caecum / appendix | 39^b^ | | 1/5 discoid resection (11/52), remaining segmental resection (42/52) |
| Chapron *et al.*  (2006) | France | Retrospective (from cohort) | 31.8 ± 5.6 | 30  sigma | 18  caecum /  ileocecal junction / appendix | 48 | 0 | Complete surgical excision |
| Dobò *et al.*  (2023) | Hungary | Prospective  (RCT) | Gr1 35±5  Gr2 34±5 | 16  sigma | 8  caecum | 24^b^ | | Resection |
| Dousset *et al.*  (2010) | France | Prospective (cohort) | 32 ± 5 | 23  sigma | 13  caecum / appendix | 36 | 8 | Resection |
| Faccioli *et al.*  (2010) | Italy | Prospective (cohort) | 31.9 (21-43)^a^ | 13  sigma | 3  caecum | 16 | 0 | 22/65 bowel resections,  10 refuse major surgery,  the remaining:  superficial excision, serosal shaving, full-thickness disk excision and segmental resection;  all histologically confirmed. |
| Fleisch *et al.*  (2005) | Germany | Retrospective (case series) | 31.9 ± 3.5 | 4  sigma | 1  caecum | 5 | 0 | Resection |
| Hernández Gutiérrez *et al.*  (2019) | Spain | Retrospective (from cohort) | 36.3 ± 5.6 | 5  sigma | 9  caecum / appendix | 14^b^ | | Resection |
| Ianieri *et al.*  (2024) | Italy | Retrospective (from cohort) | 38.4 ± 5.9 | 24  sigma + rectum or only sigma | 2  ileocecal junction + rectum | 26 | 0 | Resection |
| Keckstein *et al.*  (2005) | Austria | Prospective (cohort) | 33 (22-54)^a^ | 32  sigma | 20  caecum / appendix | 52^b^ | | Resection |
| Marcellin *et al.*  (2019) ^d^ | France | Retrospective (from cohort) | Gr1 33.1±5.7 G2 34.2±5.1 | 73  sigma | 45  ileocecal junction / appendix | 118 | 32 | Resection |
| Martinez-Serrano *et al.*  (2015) | Spain | Prospective (cohort) | Gr1 54.6^c^  Gr2 48.3^c^ | 25  sigma /  left hemicolon | 14  right hemicolon | 39^b^ | | Resection |
| Minelli *et al.*  (2009) ^e^ | Italy | Prospective (cohort) | 32 (22-47)^a^ | 35  sigma | 0^f^ | 35^f^ | | Resection |
| Mohr *et al.*  (2005) | US | Retrospective (case series) | 35^c^ (21-56)^a^ | 12  sigma | 7  caecum / appendix | 19 | 1 | Resection |
| Pereira *et al.*  (2009) | Canada | Retrospective (from cohort) | (21-53)^a^ | 64  sigma /  descending colon | 42  terminal ileum /  caecum / appendix | 106^b^ | | Resection |
| Prystowsky *et al.*  (1988) | US | Retrospective (case series) | 42.6 **±** 4.6 | 4  sigma | 13  caecum / appendix | 17^b^ | | Resection |
| Redwine *et al.*  (1999) | US | Retrospective (from cohort) | NR | 279  sigma | 78  caecum / appendix | 375^b^ | | Not specified type of surgery but all surgically treated |
| Roman *et al.*  (2020) ^g^ | France | Retrospective (from cohort) | NR | 432  sigma | 159  caecum /  ileocecal junction / appendix | 591^b^ | | Left: 39% intestinal resections, remaining disc excision and shaving.  Right: all resection |
| Urbach *et al.*  (1998) | Canada | Retrospective (case series) | 37 ± 5.5 | 9  sigma | 9  terminal ileum /  caecum / appendix | 18^b^ | | Resection |
| Weed and Ray  (1987) | US | Retrospective (from cohort) | 32 (16-60)^a^ | 65  sigma /  descending colon | 42  terminal ileum /  caecum / appendix | 107^b^ | | 20 bowel resections (sigma), remaining lesions resection of implants |
| Yantiss *et al.*  (2001) | US | Retrospective (case series) | 44 (28-56)^a^ | 18  sigma | 11  caecum / appendix | 29^b^ | | Resection |
| Zannoni *et al.*  (2017) | Italy | Prospective (cross-sectional) | 37 **±** 5.3 | 9  sigma | 2  ileocecal junction | 11^b^ | | 13/41 (31.7%) bowel resection, 28/41 (68.3%) shaving technique |

^a^ Range (min-max) or (min-max) only.

^b^ Articles in which it is not expressly stated whether the total lesions considered are unilateral or bilateral. Most articles do not mention bilateral lesions, but only multiple resections without specifying the location, making it impossible to classify bilateral lesions.

^c^ Median.

^d^ Some patients previously identified in the study by Dousset *et al.* (2010) may also be included in this report, but the former is a prospective study analysing patients operated for low rectal endometriosis (1995-2003), whereas Marcellin *et al.* (2019) is a retrospective study focusing on ileocecal endometriosis (1995-2015).

^e^ The population included in the study by Minelli *et al.* (2009) may also encompass patients described in the study by Faccioli *et al.* (2009). However, the former covers a longer study period (2002–2006 vs. 2005–2007) and includes patients from several hospitals (Sacro Cuore Hospital in Verona, the Catholic University of the Sacred Heart in Rome, and Campobasso).

^f^ Nine appendicular lesions were not considered as right lesions because they were associated with sigmoid resections. In addition, the total number of multiple bowel resections was 22, but it was not specified whether they were bilateral or unilateral.

^g^ This article may include some patients previously reported by Abo *et al.* 2018, but with a slightly different study period (Jun 2009-Dec 2015 vs Oct 2009-May 2019).

SD: Standard Deviation

NR: Not Reported

RCT: Randomized Clinical Trial
