# Supplementary material for: Lateral distribution of endometriotic lesions: the anatomical recesses hypothesis. A systematic review and meta-analysis
Source: Hum Reprod Open. 2025 Oct 24;2026(1):hoaf064. doi: 10.1093/hropen/hoaf064 (PMC12816922; doi:10.1093/hropen/hoaf064)
Supplement: hoaf064_Supplementary_Data [file hoaf064_supplementary_data.zip › Supplementary Table S5.docx]

**Supplementary Table S5.** Main characteristics of the selected studies evaluating the lateral distribution of endometriotic thoracic lesions.

| **Author,** **year** | **Country** | **Study design** | **Age**  **(mean ± SD)** | **Principal site of thoracic endometriosis** | **No of patients with left lesion** | **No of patients with right lesion** | **Sum of patients with unilateral lesion** | **No of patients with bilateral lesion** | **Other thoracic localisations** |
| --- | --- | --- | --- | --- | --- | --- | --- | --- | --- |
| Attaran *et al.*  (2013) | UK | Retrospective (from cohort) | 35 (26-42)^a^ | diaphragm | 0 | 11 | 11 | 1 | 5 with pleural involvement also |
| Bagan *et al.*  (2003) | France | Retrospective (case series) | 37.2 (21-44)^a^ | diaphragm and lung | 1 | 8 | 9 | 1 | 5 diaphragm, 4 lung |
| Bobbio *et al.* ^c^  (2024) | France | Retrospective (case series) | 36 ^b^  (27-58)^a^ | diaphragm | 2 | 18 | 20 | 0 |  |
| Campisi *et al.*  (2022) | Italy | Retrospective (from cohort) | 27.8 (21-34)^a^ | diaphragm and lung | 3 | 21 | 24 | 0 | 19 with lung involvement also |
| Ceccaroni *et al.*  (2021) | Italy | Retrospective (case series) | 35 (30-38)^a^ | diaphragm | 2 | 196 | 198 | 17 | 8 with pleural involvement also |
| Ciriaco *et al.*  (2009) | Italy | Retrospective (case series) | 32 ± 11 | diaphragm and lung | 0 | 10 | 10 | 0 | 5 with lung involvement also |
| Duyos *et al.*  (2013) | Spain | Retrospective (case series) | 35.4 (26-42)^a^ | diaphragm, lung, pleura | 0 | 5 | 5 | 0 | 1 lung and diaphragm, 1 diaphragm, 1 pleura, 1 lung  1 pleura and lung |
| Ezemba *et al.*  (2021) | Nigeria | Retrospective (case series) | 32 (24-45)^a^ | diaphragm, lung, pleura | 0 | 21 | 21 | 2 | 12 diaphragm/pleura,  remaining lung |
| Flieder *et al.*  (1998) | US | Retrospective (case series) | 36^b^  (27-74)^a^ | lung and pleura | 2 | 7 | 9 | 0^d^ | 5 lung, 4 pleura |
| Furuta *et al.*  (2018) | Japan | Retrospective (case series) | 36 (26-42)^a^ | diaphragm and lung | 0 | 9 | 9 | 0 | 5 diaphragm, 4 lung,  3 with pleural involvement also |
| Ghigna *et al.*  (2015) | France | Retrospective (case series) | 32.5 ± 8.3 | diaphragm | 0 | 18 | 18 | 0 | 14 diaphragm, 4 pleura, 6 with lung involvement also |
| Haga *et al.*  (2014) | Japan | Retrospective (from cohort) | 38.6 ± 5.7 | diaphragm, lung, pleura | 1 | 91 | 92 | 0 |  |
| Härkki *et al.*  (2010) | Finland | Retrospective (case series) | 35 (29-40)^a^ | diaphragm | 0 | 6 | 6 | 0 | 1 with lung involvement also |
| Inoue *et al.*  (2015) | Japan | Retrospective (case series) | 40.2 ± 9 | diaphragm | 1 | 11 | 12 | 1 | 1 with pleural involvement also |
| Korom *et al.*  (2004) | Switzerland | Retrospective (case series) | 34 ± 3.5 | diaphragm | 0 | 3 | 3 | 0 | 1 with lung involvement also |
| Legras *et al.*  (2014) ^e^ | France | Retrospective (from cohort) | 36.5 (31-42)^a^ | diaphragm | 1 | 51 | 52 | 2 | 30 with pleural involvement also |
| Leong *et al.*  (2006) | UK | Retrospective (case series) | 37 (34-40)^a^ | diaphragm | 0 | 4 | 4 | 0 | 1 with pleural involvement also |
| Marshall *et al.*  (2005) | US | Retrospective (case series) | 35 | diaphragm | 0 | 8 | 8 | 0 | 2 with pleural and 2 with lung involvement also |
| Nezhat *et al.*  (1998) | US | Retrospective (case series) | 31 (18-43)^a^ | diaphragm | 2 | 14 | 16 | 8 |  |
| Nezhat *et al.*  (2014) | US | Retrospective (case series) | 37.7 | diaphragm, lung, pleura | 5 | 17 | 22 | 3 | 16 with pleural and 10 with lung involvement also |
| Ochi *et al.*  (2022) | Japan | Retrospective (from cohort) | 41^b^  (22-53)^a^ | diaphragm and pleura | 1 | 159 | 160 | 0 | 150 with pleural involvement also |
| Pagano *et al.*  (2023) | Switzerland | Prospective (cohort) | 34 (19-49)^a^ | diaphragm | 5 | 60 | 65 | 0 |  |
| Piriyev and Romer  (2024) | Germany | Retrospective (from cohort) | Gr1 32.5±6.7  Gr2 31.8±6.9 | diaphragm | 6 | 74 | 80 | 21 |  |
| Redwine *et al.*  (2002) | US | Retrospective (case series) | Gr1 30.7  Gr2 35.0 | diaphragm and pleura | 0 | 7 | 7 | 1 |  |
| Rousset *et al.*  (2016) | France | Retrospective (from cohort) | 32 (24-41)^a^ | diaphragm | 0 | 23 | 23 | 0 |  |
| Rousset-Jablonski *et al.*  (2011) | France | Retrospective (from cohort) | 31.8 ± 8.7 | diaphragm and pleura | 1 | 45 | 46 | 3 |  |
| Tulandi *et al.*  (2018) | Canada | Prospective (case-control study) | 35.6 ± 6.6 | pleura | 0 | 12 | 12 | 0 | 2 with lung involvement also |
| Vercellini *et al.*  (2007) | Italy | Retrospective (case series) | 31 (18-50)^a^ | diaphragm | 1 | 5 | 6 | 1 |  |
| Viti *et al.*  (2020) | Italy | Retrospective (case series) | 38 | diaphragm | 1 | 20 | 21 | 1 | 4 with pleural and 3 with lung involvement also |
| Wetzel *et al.*  (2021) | France | Retrospective (from cohort) | 32 (24-46)^a^ | diaphragm | 1 | 45 | 46 | 4 | 4 with pleural and 3 with lung involvement also |

^a^ Range (min-max).

^b^ Median.
^c^ Some overlap with the population described by Legras *et al.* (2014) cannot be excluded, as both studies may have drawn from the same initial cohort. However, only patients with diaphragmatic hernia were selected in the present study (Bobbio *et al.,* 2024).

^d^ One case of bilateral decidualised thoracic endometriosis due to ongoing pregnancy was excluded.

^e^ Some of the patients analysed in this study (Legras *et al.,* 2014) may have already been reported in an earlier publication by the same group (Rousset-Jablonski *et al.,* 2011), although the recruitment periods differ slightly, and the former covers a longer timeframe (Nov 2000-Sept 2011 vs. Jul 2000-Jan 2009).

SD: Standard Deviation

MRI: Magnetic Resonance Imaging
