# Supplementary material for: Lateral distribution of endometriotic lesions: the anatomical recesses hypothesis. A systematic review and meta-analysis
Source: Hum Reprod Open. 2025 Oct 24;2026(1):hoaf064. doi: 10.1093/hropen/hoaf064 (PMC12816922; doi:10.1093/hropen/hoaf064)
Supplement: hoaf064_Supplementary_Data [file hoaf064_supplementary_data.zip › Supplementary Table S6.docx]

**Supplementary Table S6.** Main characteristics of the selected studies evaluating the lateral distribution of endometriotic inguinal lesions.

| **Author, year** | **Country** | **Study design** | **Age**  **(mean ± SD)** | **No of patients with left lesion** | **No of patients with right lesion** | **Sum of patients with unilateral lesion** | **No of patients with bilateral lesion** | **Type of surgery** |
| --- | --- | --- | --- | --- | --- | --- | --- | --- |
| Apostolidis *et al.*  (2009) | Greece | Retrospective (case series) | 38 ± 2.9 | 0 | 3 | 3 | 0 | Radical excision of the lesion en bloc with the extraperitoneal portion of the round ligament |
| Arakawa *et al.*  (2019) | Japan | Retrospective (case series) | 37.2± 6.7 | 5 | 13 | 18 | 2 | Complete resection of the intra- and extraperitoneal portion of round ligament in 6 (3 right, 2 bilateral, 1 left). The remaining diagnosed with MRI |
| Candiani *et al.*  (1991) | Italy | Retrospective (case series) | 31 (22-46)^a^ | 0 | 5 | 5 | 1 | Complete excision of lesion of round ligament |
| Chou *et al.*  (2023) | Taiwan | Retrospective (case series) | 41±4 | 0 | 2 | 2 | 0 | Excision of lesion and part of the round ligament |
| Dormandy  (1969) | UK | Retrospective (case series) | 38,40,49 | 0 | 3 | 3 | 0 | Complete excision of lesion of round ligament |
| Fedele *et al.*  (2007) | Italy | Retrospective (case series) | 36 (28-43)^a^ | 1 | 4 | 5 | 0 | Radical excision of the lesion en bloc with the extraperitoneal portion of the round ligament |
| Haghgoo *et al.*  (2024) | Iran | Retrospective (case series) | 36.3 ± 6.8 | 1 | 6 | 7 | 0 | Surgical excision of the inguinal mass in 4, remaining ultrasound and/or MRI diagnosis |
| Jimenez and  Miles  (1960) | US | Retrospective (case series) | 49,34,39,36 | 0 | 4 | 4 | 0 | Complete excision of lesion of round ligament |
| Kapan *et al.*  (2005) | Turkey | Retrospective (case series) | 44 ±3.6 | 1 | 2 | 3 | 0 | Excision of lesion and round ligament |
| Li *et al.*  (2021) | China | Retrospective (case series) | 38 (32-53)^a^ | 1 | 8 | 9 | 1 | Radical excision of the lesion en bloc with the extraperitoneal portion of the round ligament |
| Miranda *et al.*  (2001) | Italy | Retrospective (case series) | (32-40)^a^ | 1 | 1 | 2 | 0 | Complete excision of lesion of round ligament |
| Mongelli *et al.*  (2022) | Italy | Retrospective (case series) | 30.5 | 0 | 2 | 2 | 0 | Radical excision of the lesion en bloc with the extraperitoneal portion of the round ligament |
| Mu *et al.*  (2021) | China | Retrospective (case series) | 36 ± 4 | 0 | 2 | 2 | 1 | Radical excision of the lesion en bloc with the extraperitoneal portion of the round ligament |
| Niitsu *et al.*  (2019) | Japan | Retrospective (from cohort) | 36.6 | 3 | 25 | 28 | 0 | Wide surgical margin with excision of the round ligament |
| Pellegrini *et al.*  (1981) | Italy | Retrospective (case series) | 30-36 | 0 | 2 | 2 | 0 | Radical excision of the lesion en bloc with the extraperitoneal portion of the round ligament |
| Sun *et al.*  (2010) | China | Retrospective (case series) | 41.7 (30-52)^a^ | 1 | 8 | 9 | 0 | Complete excision of inguinal lesion in 8. Wide excision with the extraperitoneal portion of the round ligament in one |
| Wolfhagen *et al.*  (2018) | Netherlands | Retrospective (case series) | 32.5 (27-43)^a^ | 2 | 7 | 9 | 0 | Complete excision of lesion of round ligament |
| Yang *et al.*  (2010) | Korea | Retrospective (case series) | 29,43,49 | 0 | 3 | 2 | 0 | Complete excision of lesion of round ligament |

^a^ Range (min-max).

SD: Standard Deviation

MRI: Magnetic Resonance Imaging
