# Supplementary material for: Lateral distribution of endometriotic lesions: the anatomical recesses hypothesis. A systematic review and meta-analysis
Source: Hum Reprod Open. 2025 Oct 24;2026(1):hoaf064. doi: 10.1093/hropen/hoaf064 (PMC12816922; doi:10.1093/hropen/hoaf064)
Supplement: hoaf064_Supplementary_Data [file hoaf064_supplementary_data.zip › Supplementary Table S7.docx]

**Supplementary Table S7.** Risk of bias assessment for non-randomised studies reporting lateral distribution of endometriotic lesions, based on the ROBINS-I tool.

| **Author, year** | **Type of bias** | | | | | | | **Overall rating**  (low, moderate, serious, critical) |
| --- | --- | --- | --- | --- | --- | --- | --- | --- |
|  | **Bias due to confounding** | **Bias due to selection of participants** | **Bias in classification of interventions** | **Bias due to deviations from intended intervention** | **Bias due to missing data** | **Bias**  **in measurement of outcomes** | **Bias in selection of the reported result** |  |
| Abbott *et al.* (2003) | Moderate | Low | Low | Moderate | Moderate | Moderate | Low | Moderate |
| Abdalla Ribeiro *et al.* (2021) | Low | Low | Low | Low | Low | Moderate | Low | Moderate |
| Abo *et al.* (2018) | Low | Moderate | Low | Low | Moderate | Low | Low | Moderate |
| Alborzi *et al.* (2017) | Low | Low | Low | Low | Low | Low | Low | Low |
| Anaf *et al.* (2009) | Low | Low | Low | Low | Low | Low | Low | Low |
| Antonelli *et al.* (2006) | Moderate | Low | Low | Low | Low | Moderate | Moderate | Moderate |
| Ari *et al.* (2023) | Moderate | Moderate | Low | Low | Moderate | Low | Moderate | Moderate |
| Attaran *et al.* (2013) | Low | Moderate | Low | Low | Moderate | Low | Moderate | Moderate |
| Audebert *et al.* (2018) | Low | Low | Low | Low | Low | Low | Low | Low |
| Bazot *et al*. (2012) | Moderate | Moderate | Low | Low | Moderate | Low | Moderate | Moderate |
| Bhurke *et al.* (2022) | Low | Low | Low | Low | Low | Low | Low | Low |
| Bindra *et al.* (2023) | Low | Moderate | Low | Low | Moderate | Low | Low | Moderate |
| Buffeteau *et al.* (2023) | Moderate | Low | Low | Low | Moderate | Low | Moderate | Moderate |
| Campisi *et al.* (2022) | Moderate | Low | Low | Low | Low | Moderate | Moderate | Moderate |
| Ceccaroni *et al.* (2019) | Moderate | Low | Low | Low | Low | Moderate | Low | Moderate |
| Chapron *et al.* (2006) | Low | Low | Low | Low | Low | Low | Low | Low |
| Chapron *et al.* (2010) | Moderate | Low | Low | Low | Moderate | Low | Moderate | Moderate |
| Chopin *et al.* (2006) | Low | Low | Low | Low | Low | Low | Low | Low |
| Coccia *et al.* (2021) | Moderate | Low | Low | Low | Low | Moderate | Low | Moderate |
| Dafna *et al.* (2019) | Low | Moderate | Low | Low | Moderate | Low | Low | Moderate |
| Darwish *et al.* (2017) | Low | Moderate | Low | Low | Moderate | Low | Low | Moderate |
| Di Giovanni *et al.* (2023) | Low | Low | Low | Low | Low | Low | Low | Low |
| Dobò *et al.* (2023)^a^ | Low | Low | Low | Low | Low | Low | Low | Low |
| Donnez *et al.* (2002) | Moderate | Low | Low | Low | Low | Moderate | Low | Moderate |
| Dousset *et al.* (2010) | Low | Low | Low | Low | Low | Low | Low | Low |
| Ercan *et al.* (2011) | Low | Low | Low | Low | Low | Moderate | Low | Moderate |
| Ferrero *et al.* (2005) | Low | Low | Low | Low | Low | Low | Low | Low |
| Faccioli *et al.* (2009) | Low | Low | Low | Low | Low | Low | Low | Low |
| Freger *et al*. (2024) | Low | Low | Low | Low | Low | Moderate | Low | Moderate |
| Ghezzi *et al.* (2001) | Low | Low | Low | Low | Low | Moderate | Low | Moderate |
| Ghezzi *et al.* (2006) | Low | Low | Low | Low | Low | Low | Low | Low |
| Haga *et al.* (2014) | Low | Low | Low | Low | Low | Low | Low | Low |
| Harada *et al.* (2015) | Low | Low | Low | Low | Low | Low | Low | Low |
| Hernández Gutiérrez *et al.* (2019) | Low | Low | Low | Low | Low | Low | Low | Low |
| Hudelist *et al.* (2009) | Low | Low | Low | Low | Low | Moderate | Low | Moderate |
| Ianieri *et al.* (2024) | Low | Low | Low | Low | Low | Low | Low | Low |
| Keckstein *et al.* (2005) | Moderate | Moderate | Low | Low | Moderate | Low | Moderate | Moderate |
| Khan *et al.* (2013) | Low | Low | Low | Low | Low | Low | Low | Low |
| Kikuchi *et al.* (2006) | Low | Low | Low | Low | Low | Moderate | Low | Moderate |
| Kwok *et al.* (2020) | Low | Low | Low | Low | Low | Low | Low | Low |
| Lee *et al.* (2013) | Low | Low | Low | Low | Low | Low | Low | Low |
| Legras *et al.* (2014) | Low | Moderate | Low | Low | Low | Moderate | Low | Moderate |
| Liu *et al.* (2008) | Low | Moderate | Low | Low | Moderate | Low | Low | Moderate |
| Malzoni *et al.* (2016) | Low | Low | Low | Low | Low | Low | Low | Low |
| Marcellin *et al.* (2019) | Moderate | Low | Low | Low | Moderate | Moderate | Moderate | Moderate |
| Martinez-Serrano *et al.* (2015) | Low | Low | Moderate | Low | Moderate | Low | Low | Moderate |
| Matalliotaki *et al*. (2020) | Low | Moderate | Low | Low | Moderate | Low | Low | Moderate |
| Mereu *et al.* (2010) | Low | Moderate | Low | Low | Moderate | Low | Low | Moderate |
| Mereu *et al.* (2012) | Low | Low | Low | Low | Low | Low | Low | Low |
| Meuleman *et al.* (2009) | Low | Low | Low | Low | Low | Low | Low | Low |
| Minelli *et al.* (2009) | Low | Low | Low | Low | Moderate | Low | Low | Moderate |
| Moro *et al.* (2024) | Low | Low | Low | Low | Low | Low | Low | Low |
| Nicolaus *et al.* (2020) | Low | Moderate | Low | Low | Moderate | Low | Low | Moderate |
| Niitsu *et al.*  (2019) | Moderate | Low | Low | Low | Moderate | Moderate | Moderate | Moderate |
| Ochi *et al.* (2022) | Low | Low | Low | Low | Low | Low | Low | Low |
| Pagano *et al.* (2023) | Low | Low | Low | Low | Low | Low | Low | Low |
| Parazzini (2003) | Moderate | Low | Low | Low | Moderate | Moderate | Moderate | Moderate |
| Pereira *et al.* (2009) | Low | Low | Low | Low | Moderate | Low | Low | Moderate |
| Piriyev and Romer (2024) | Low | Low | Low | Low | Low | Moderate | Low | Moderate |
| Porpora *et al.* (2014) | Low | Moderate | Low | Low | Moderate | Low | Low | Moderate |
| Qiu *et al.* (2023) | Moderate | Low | Low | Low | Moderate | Moderate | Low | Moderate |
| Redwine *et al.* (1999) | Low | Low | Low | Low | Low | Low | Low | Low |
| Roman *et al.* (2010) | Low | Low | Low | Low | Low | Moderate | Low | Moderate |
| Roman *et al.* (2020) | Low | Low | Low | Low | Low | Low | Low | Low |
| Rousset *et al.* (2016) | Moderate | Low | Low | Low | Moderate | Moderate | Low | Moderate |
| Rousset-Jablonski *et al.* (2011) | Moderate | Moderate | Low | Low | Low | Moderate | Low | Moderate |
| Serracchioli *et al.* (2008) | Moderate | Moderate | Low | Low | Moderate | Moderate | Low | Moderate |
| Serracchioli *et al.* (2014)^a^ | Low | Low | Low | Low | Low | Low | Low | Low |
| Serracchioli *et al.* (2015) | Low | Low | Low | Low | Low | Low | Low | Low |
| Sesti *et al.* (2009)^a^ | Low | Low | Low | Low | Low | Low | Low | Low |
| Signorile *et al.* (2022) | Low | Serious | Moderate | Low | Serious | Moderate | Moderate | Serious |
| Sillou *et al.*  (2015) | Low | Moderate | Low | Low | Moderate | Low | Low | Moderate |
| Soriano *et al.* (2011) | Low | Low | Low | Low | Low | Moderate | Low | Low |
| Stoppa *et al.* (2023) | Moderate | Low | Low | Low | Moderate | Low | Moderate | Moderate |
| Tulandi *et al.* (2018) | Low | Low | Low | Low | Low | Low | Low | Low |
| Uccella *et al.* (2014) | Low | Low | Low | Low | Low | Moderate | Low | Moderate |
| Weed and Ray (1987) | Low | Serious | Moderate | Low | Moderate | Low | Low | Serious |
| Wang *et al.*  (2015) | Low | Moderate | Low | Low | Moderate | Low | Low | Moderate |
| Wetzel *et al.* (2021) | Moderate | Moderate | Low | Low | Moderate | Low | Low | Moderate |
| Yamada *et al.* (2022) | Low | Low | Low | Low | Low | Low | Low | Low |
| Yu *et al.* (2015) | Low | Low | Low | Low | Low | Low | Low | Low |
| Yuan *et al*. (2014) | Low | Low | Low | Low | Low | Moderate | Low | Moderate |
| Zannoni *et al.* (2017) | Low | Low | Low | Low | Low | Low | Low | Low |

^a^ The study design was a prospective randomised controlled trial. However, since randomisation was independent of laterality, risk of bias was assessed as for a non-randomised study.
