# Supplementary material for: Lateral distribution of endometriotic lesions: the anatomical recesses hypothesis. A systematic review and meta-analysis
Source: Hum Reprod Open. 2025 Oct 24;2026(1):hoaf064. doi: 10.1093/hropen/hoaf064 (PMC12816922; doi:10.1093/hropen/hoaf064)
Supplement: hoaf064_Supplementary_Data [file hoaf064_supplementary_data.zip › Supplementary Table S8.docx]

**Supplementary Table S8.** Risk of bias assessment for case series reporting lateral distribution of endometriotic lesions.

| **Author, year** | **Selection** | **Ascertainment** | | **Causality** | | **Reporting** | **Overall rating (0-6)** |
| --- | --- | --- | --- | --- | --- | --- | --- |
|  | Does the patient(s) represent(s) the whole experience of the investigator or is the selection method unclear to the extent  that other patients with similar presentation may not have been reported? | Was the exposure adequately ascertained? | Was the outcome adequately ascertained? | Were other alternative causes that may explain the observation ruled out? | Was follow-up long enough for outcomes to occur? | Is the case(s) described with sufficient details to allow other investigators to replicate the research or to allow practitioners make  inferences related to their own practice? |  |
| Al-Fozan and Tulandi (2003) | 1 | 1 | 1 | 0 | 0 | 1 | 3 |
| Al-Khawaja *et al.* (2008) | 1 | 1 | 1 | 1 | 1 | 1 | 6 |
| Apostolidis *et al.*  (2009) | 0 | 1 | 0 | 0 | 0 | 1 | 2 |
| Arakawa *et al.*  (2019) | 1 | 1 | 1 | 0 | 1 | 1 | 5 |
| Araujo *et al.* (2021) | 1 | 1 | 1 | 1 | 1 | 1 | 6 |
| Azioni *et al.* (2010) | 1 | 1 | 1 | 1 | 1 | 1 | 6 |
| Bagan *et al.* (2003) | 0 | 1 | 1 | 0 | 0 | 0 | 2 |
| Bailey *et al.* (1994) | 1 | 1 | 1 | 0 | 0 | 1 | 4 |
| Bazi *et al.* (2007) | 1 | 1 | 0 | 0 | 0 | 1 | 3 |
| Bobbio *et al.* (2024) | 1 | 1 | 1 | 1 | 1 | 1 | 6 |
| Bosev *et al.* (2009) | 1 | 1 | 1 | 0 | 1 | 1 | 5 |
| Bouaziz *et al.* (2017) | 1 | 1 | 1 | 0 | 1 | 1 | 5 |
| Candiani *et al.* (1991) | 1 | 1 | 1 | 0 | 1 | 1 | 5 |
| Ceccaroni *et al.* (2021) | 1 | 1 | 1 | 1 | 1 | 1 | 6 |
| Chapron *et al.* (2001) | 1 | 1 | 1 | 1 | 1 | 1 | 6 |
| Chou *et al.* (2023) | 1 | 1 | 1 | 0 | 1 | 1 | 5 |
| Ciavattini *et al*. (2004) | 1 | 1 | 1 | 1 | 1 | 1 | 6 |
| Ciriaco *et al*. (2009) | 1 | 1 | 1 | 0 | 1 | 1 | 5 |
| Dormandy (1956) | 0 | 1 | 1 | 0 | 1 | 1 | 4 |
| Duyos *et al.* (2014) | 0 | 1 | 1 | 0 | 1 | 1 | 4 |
| Ezemba *et al.* (2021) | 1 | 1 | 0 | 0 | 1 | 1 | 4 |
| Fedele *et al.* (2007) | 1 | 1 | 1 | 0 | 1 | 1 | 5 |
| Fleisch *et al.* (2005) | 0 | 1 | 1 | 0 | 1 | 1 | 4 |
| Flieder *et al.* (1998) | 1 | 1 | 1 | 0 | 0 | 1 | 4 |
| Frenna *et al.* (2007) | 1 | 1 | 1 | 0 | 1 | 1 | 5 |
| Furuta *et al.* (2018) | 1 | 1 | 1 | 1 | 1 | 1 | 6 |
| Ghigna *et al.* (2015) | 0 | 1 | 1 | 0 | 1 | 1 | 4 |
| Haghgoo *et al.* (2024) | 1 | 1 | 1 | 0 | 1 | 1 | 5 |
| Härkki *et al.* (2010) | 1 | 1 | 1 | 0 | 1 | 1 | 5 |
| Hung *et al.* (2020) | 1 | 1 | 1 | 0 | 1 | 1 | 5 |
| Inoue *et al.* (2015) | 1 | 1 | 1 | 0 | 1 | 1 | 5 |
| Jenkins *et al.* (1986) | 0 | 1 | 1 | 0 | 1 | 1 | 4 |
| Jimenez and Miles (1960) | 0 | 1 | 1 | 0 | 1 | 1 | 4 |
| Kapan *et al.* (2005) | 1 | 1 | 1 | 0 | 1 | 1 | 5 |
| Knabben *et al.* (2015) | 1 | 1 | 1 | 0 | 1 | 1 | 5 |
| Korom *et al.* (2004) | 1 | 1 | 1 | 1 | 1 | 1 | 6 |
| Kovoor *et al.* (2011) | 1 | 1 | 1 | 0 | 1 | 1 | 5 |
| Langebrekke and Qvigstad (2011) | 1 | 1 | 1 | 0 | 1 | 1 | 5 |
| Langmade (1975) | 0 | 1 | 1 | 0 | 0 | 1 | 3 |
| Leong *et al.* (2006) | 1 | 1 | 1 | 1 | 1 | 1 | 6 |
| Li *et al.* (2021) | 1 | 1 | 1 | 0 | 1 | 1 | 5 |
| Li *et al*. (2024) | 1 | 1 | 1 | 0 | 1 | 1 | 5 |
| Marshall *et al.* (2005) | 1 | 1 | 1 | 0 | 1 | 1 | 5 |
| Matalliotakis *et al.* (2017) | 1 | 1 | 1 | 0 | 1 | 1 | 5 |
| Miranda *et al.* (2001) | 1 | 1 | 1 | 0 | 1 | 1 | 5 |
| Miranda-Mendoza *et al.* (2012) | 1 | 1 | 1 | 0 | 1 | 1 | 5 |
| Mongelli *et al.* (2022) | 1 | 1 | 1 | 1 | 1 | 1 | 6 |
| Mohr *et al.* (2005) | 1 | 1 | 1 | 0 | 1 | 1 | 5 |
| Mu *et al.* (2021) | 1 | 1 | 1 | 0 | 1 | 1 | 5 |
| Nezhat *et al.* (1996) | 1 | 1 | 1 | 0 | 1 | 1 | 5 |
| Nezhat *et al.* (1998) | 1 | 1 | 1 | 0 | 1 | 1 | 5 |
| Nezhat *et al.* (2014) | 1 | 1 | 1 | 0 | 1 | 1 | 5 |
| Özyer *et al.* (2013) | 1 | 1 | 1 | 0 | 1 | 1 | 5 |
| Pellegrini *et al.* (1981) | 1 | 1 | 1 | 0 | 0 | 1 | 4 |
| Pérez Utrilla Pérez *et al.* (2009) | 1 | 1 | 1 | 0 | 1 | 1 | 5 |
| Prefumo *et al.* (2002) | 1 | 1 | 1 | 0 | 1 | 1 | 5 |
| Prystowsky *et al.* (1988) | 1 | 1 | 1 | 0 | 1 | 1 | 5 |
| Pugliese *et al.* (2006) | 1 | 1 | 1 | 0 | 1 | 1 | 5 |
| Redwine (2002) | 1 | 1 | 1 | 1 | 1 | 1 | 6 |
| Song *et al.* (2016) | 1 | 1 | 1 | 0 | 1 | 1 | 5 |
| Sun *et al.* (2010) | 0 | 1 | 1 | 0 | 1 | 1 | 4 |
| Sznurkowski and Emerich (2008) | 1 | 1 | 1 | 0 | 1 | 1 | 5 |
| Ulukus *et al.* (2012) | 1 | 1 | 1 | 0 | 1 | 1 | 5 |
| Urbach *et al.* (1998) | 1 | 1 | 1 | 0 | 1 | 1 | 5 |
| Vercellini *et al.* (2007) | 1 | 1 | 1 | 1 | 1 | 1 | 6 |
| Vercellini *et al.* (2000b) | 1 | 1 | 1 | 0 | 1 | 1 | 5 |
| Vercellini *et al.* (1998) | 1 | 1 | 1 | 0 | 1 | 1 | 5 |
| Viti *et al.* (2020) | 1 | 1 | 1 | 1 | 1 | 1 | 6 |
| Wolfhagen *et al.* (2018) | 1 | 1 | 1 | 1 | 0 | 1 | 5 |
| Yang *et al.* (2010) | 1 | 1 | 1 | 0 | 1 | 1 | 5 |
| Yantiss *et al.* (2001) | 1 | 1 | 1 | 1 | 0 | 1 | 5 |
